# Ab Initio Molecular Cavity Quantum Electrodynamics Simulations Using Machine Learning Models

**DOI:** 10.1021/acs.jctc.3c00137

**Published:** 2023-03-31

**Authors:** Deping Hu, Pengfei Huo

**Affiliations:** Department of Chemistry, University of Rochester, Rochester, New York 14627, United States

## Abstract

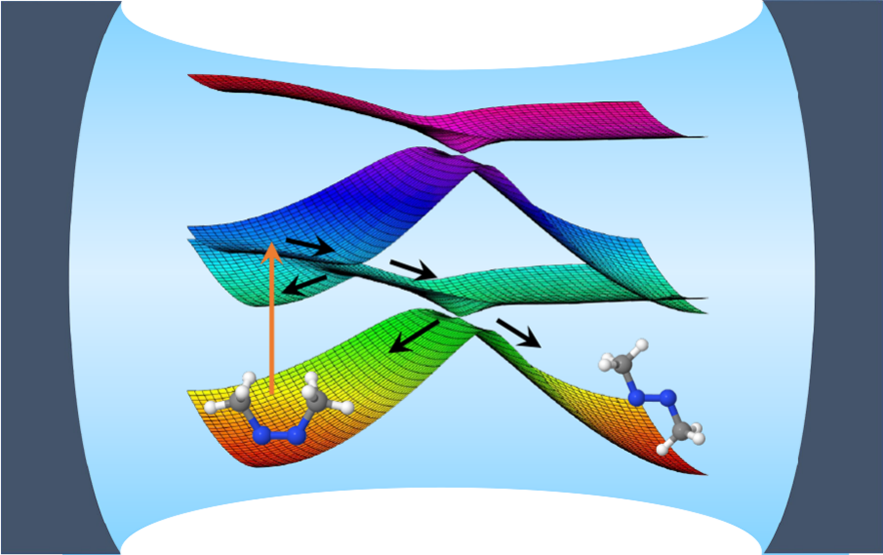

We present a mixed quantum-classical simulation of polariton dynamics for molecule–cavity hybrid systems. In particular, we treat the coupled electronic–photonic degrees of freedom (DOFs) as the quantum subsystem and the nuclear DOFs as the classical subsystem and use the trajectory surface hopping approach to simulate non-adiabatic dynamics among the polariton states due to the coupled motion of nuclei. We use the accurate nuclear gradient expression derived from the Pauli–Fierz quantum electrodynamics Hamiltonian without making further approximations. The energies, gradients, and derivative couplings of the molecular systems are obtained from the on-the-fly simulations at the level of complete active space self-consistent field (CASSCF), which are used to compute the polariton energies and nuclear gradients. The derivatives of dipoles are also necessary ingredients in the polariton nuclear gradient expression but are often not readily available in electronic structure methods. To address this challenge, we use a machine learning model with the Kernel ridge regression method to construct the dipoles and further obtain their derivatives, at the same level as the CASSCF theory. The cavity loss process is modeled with the Lindblad jump superoperator on the reduced density of the electronic–photonic quantum subsystem. We investigate the azomethane molecule and its photoinduced isomerization dynamics inside the cavity. Our results show the accuracy of the machine-learned dipoles and their usage in simulating polariton dynamics. Our polariton dynamics results also demonstrate the isomerization reaction of azomethane can be effectively tuned by coupling to an optical cavity and by changing the light–matter coupling strength and the cavity loss rate.

## 1 Introduction

When coupling molecules with quantized radiation modes inside an optical cavity, a set of new photon–matter hybrid states are created due to the coupling between the molecules and the quantized radiation field.^1−7^ These new created hybrid states, which are commonly referred to as polaritons, have been shown to facilitate new chemical reactivities and selectivities.^1,6,8−10^ Theoretical investigations play a crucial role in understanding the fundamental limit and basic principles in this emerging field,^5,6,11−17^ as these polariton chemical reactions often involve a rich dynamical interplay among the electronic, nuclear, and photonic degrees of freedom (DOFs).

Recently, various theoretical methods have been developed or extended to directly simulate the polariton dynamics. These include the full quantum dynamics simulations,^18−21^ mixed-quantum-classical (MQC) dynamics,^12,13,22−25^ and non-adiabatic dynamics based on the mapping formalism.^6,26^ Among them, the MQC dynamics methods describe the electronic–photonic DOFs quantum mechanically and treat the nuclear DOFs classically; hence, they well balance the computational cost and accuracy of the dynamics. Thus, the MQC methods, including the Ehrenfest and trajectory surface hopping (TSH) methods, have been widely used in the non-adiabatic polariton dynamics recently.^11−17,23−28^

In the propagation of the polariton dynamics with the MQC methods, besides the energies of the electron–photon hybrid states, we need to derive the nuclear gradients and the couplings between these states, where the derivatives of molecular dipoles (including permanent dipoles and transition dipoles) are the key ingredients.^25,26^ For some model systems with well-defined diabatic electronic states, the dipoles of/between these diabatic states can be set to constants.^23,28^ For model systems with adiabatic electronic states, the dipoles of/between these adiabatic states can be calculated through the discrete variable representation (DVR).^25,26^ Evaluating the derivatives of molecular dipoles remains a theoretical bottleneck for simulating *ab initio* polariton quantum dynamics. These derivatives are neither readily available for most electronic structure methods nor computationally cheap to obtain.

Recent polariton quantum dynamics simulations are focusing on obtaining these expensive derivatives using semiempirical electronic structure methods. For example, Zhang et al. derived the derivative of transition dipoles at the AM1/CIS level of theory^22^ and applied it to the TSH simulations of the stilbene molecule coupled to the cavity based on the Jaynes–Cummings (JC)^29^ and Tavis–Cummings (TC) models. Fregoni et al. derived the derivative of transition dipoles at the AM1/FOMO-CI level and performed the TSH simulations for azobenzene molecule based on the Rabi-type model (that excludes the permanent dipole moment and dipole self-energy).^13,15,27^ Groenhof et al. approximated transition dipoles by a first-order Taylor expansion around the ground state equilibrium and obtained the derivatives through least-squares fitting with a large number geometrical conformations.^12,14,16,17^ The lack of accurate derivatives on dipoles (at the correlated wave function level of theory) had become the major theoretical bottleneck in performing *ab initio* on-the-fly simulations of polariton chemistry. To the best of our knowledge, there is no previous work on using the correlated wave function level of theory to perform *ab initio* on-the-fly quantum dynamics simulations of polariton chemistry with the rigorous nuclear gradient.^25^

In this work, we construct the dipoles (including permanent and transition dipoles) and their derivatives for realistic molecules using machine learning techniques.^30−34^ We apply the machine-learned dipoles and derivatives to *ab initio* on-the-fly polariton dynamics simulations of a realistic polyatomic molecular system, azomethane, coupled to an optical cavity. We use the accurate nuclear gradient expression derived from the Pauli–Fierz (PF) quantum electrodynamics Hamiltonian without making further approximations.^25^ The energies, gradients, and derivative couplings of the molecular systems are obtained from the on-the-fly simulations at the level of complete active space self-consistent field (CASSCF), which are used to compute the polariton energies and nuclear gradients. The derivatives of dipoles are obtained from the machine learning model, which is trained with the data obtained from the CASSCF level of electronic structure calculations. The cavity loss process is modeled with the Lindblad jump superoperator on the reduced density of the electronic–photonic quantum subsystem. Our results show the accuracy of the machine-learned dipoles and their usage in simulating polariton dynamics.

We perform the TSH simulations to investigate the photoisomerization reaction inside the cavity. The photoinduced non-adiabatic dynamics process of azomethane has been widely investigated.^35−39^ Since azomethane is the simplest azoalkane and has rich dynamics (i.e., photoisomerization and photodissociation), it is also often used as a model molecular system to test the performance of the non-adiabatic dynamics methods^40^ or the electrical structure methods^41^ in the non-adiabatic dynamics simulations. Thus, we adopt azomethane in this work to see how the light–matter coupling can affect the photoinduced reaction of the molecular system. Our polariton dynamics results also demonstrate the isomerization reaction of azomethane can be effectively tuned by coupling to an optical cavity. The machine learning model developed in this work illustrates its potential applications in the polariton dynamics simulations, where we can in principle construct the derivatives of dipoles for molecules with any electronic structure method if that method can provide the dipoles. This development paves the way toward simulating complex molecular systems inside an optical cavity.

## 2 Theoretical Approaches

### 2.1 Quantum Electrodynamics Hamiltonian

The Pauli–Fierz (PF) QED Hamiltonian for one molecule coupled to a quantized radiation field inside an optical cavity is expressed as
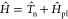
1where *T̂*_n_ represents the nuclear kinetic energy operator. Further, *Ĥ*_pl_ is commonly referred to as the polariton Hamiltonian^3,42^ and is defined as
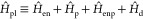
2where *Ĥ*_en_ is the electronic Hamiltonian that describes electron–nucleus interactions. In addition, *Ĥ*_p_, *Ĥ*_enp_, and *Ĥ*_d_ represent the photonic Hamiltonian, electronic–nuclear–photonic interactions, and the dipole self-energy (DSE) term, respectively.

The electronic–nuclear potential *Ĥ*_en_, which describes the common molecular Hamiltonian (excluding the nuclear kinetic energy), is described as follows
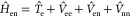
3The above expression includes electronic kinetic energy *T̂*_e_, electron–electron interaction *V̂*_ee_, electron–nucleus interaction *V̂*_en_, and nucleus–nucleus interaction *V̂*_nn_. Modern electronic structure theories have been developed around solving the eigenvalue problem of *Ĥ*_en_, providing the following electronically adiabatic energy and its corresponding state
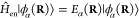
4Here, |*ϕ*_*α*_(**R**)⟩ represents the αth many-electron adiabatic state for a given molecular system, with the adiabatic energy *E*_*α*_(**R**).

For clarity, we restrict our discussions to the cavity with only one photonic mode, and all the formulas presented here can be easily generalized into a more realistic, many-mode cavity. The photonic Hamiltonian is written as
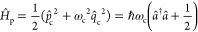
5where  and  are photon field operators, *â*^†^ and *â* are the photonic creation and annihilation operators, respectively, and ω_c_ is the photon frequency inside the cavity.

The light–matter coupling term *Ĥ*_enp_ (electronic–nuclear–photonic interactions) under the dipole gauge (and the long wavelength approximation) is expressed as
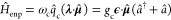
6where **λ** = λ·**ϵ** characterizes the cavity photon field strength and **ϵ** is the direction of the field polarization, which can be written as
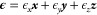
7where ***x***, ***y***, and ***z*** are the unit vectors in the *X*, *Y*, and *Z* directions, respectively. These directions are related to the cavity structure. For convenience, we also use these directions to define reference coordinate systems for all initial nuclear geometries; see section 4.2 (Figure 2a) for details. The cavity field strength is determined by the volume of the cavity as , where  is the permittivity inside the cavity and  is the effective quantization volume inside the cavity. Another commonly used light–matter coupling strength is characterized as . Further, the total dipole operator of both electrons and nuclei is defined as
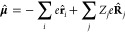
8where −*e* is the charge of the electron and *Z*_*j*_*e* is the charge of the *j*th nucleus. Finally, the dipole self-energy (DSE) term is expressed as
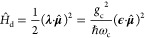
9

For the molecule–cavity hybrid system, we use the convenient photon-dressed electronic adiabatic states
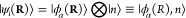
10where quantum number *i* ≡ {α, *n*} indicates both the adiabatic electronic state of the molecule and the Fock state. We refer to |*ψ*_*i*_(**R**)⟩ as the adiabatic-Fock state in this work. Note that we have introduced a shorthand notation in eq 10, which will be used throughout the rest of this paper. This is one of the most straightforward choices of basis for the hybrid system because of the readily available adiabatic electronic information (e.g., wave functions, energies, and the dipole matrix) from electronic structure calculations.

With the adiabatic-Fock state basis |*ϕ*_*ν*_(**R**), *n*⟩ and |*ϕ*_*γ*_(**R**), *m*⟩ introduced in eq 10, the matrix elements of all terms in eq 2 can be explicitly expressed as follows (using the properties of creation and annihilation operators of photonic DOF)
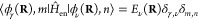
11a
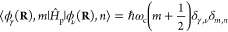
11b
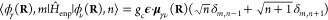
11c
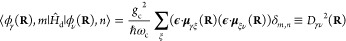
11dwhere *E*_*ν*_(**R**) and **μ**_*γν*_(**R**) explicitly depend on nuclear position **R**. A detailed derivation of these expressions can be found in ref (26). In eq 11d, *D*_*γν*_^2^ denotes the matrix elements of DSE and the sum ∑_ξ_ in the matrix element of *Ĥ*_d_ runs over the adiabatic states |*ϕ*_*ξ*_(**R**)⟩ considered in the calculation (as opposed to including all possible adiabatic states of the molecule). Note that this effectively projects **μ̂** inside the DSE term within the matter subspace, with the projection operator . This matter state truncation scheme^43,44^ makes sure all operators are properly confined in the same truncated electronic subspace^44^ in order to generate consistent and meaningful results.

In eq 11, we have used the matrix element of the dipole operator **μ̂** (eq 8) under the adiabatic representation
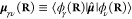
12The total dipole operator **μ̂** is a vector that can be projected into the three-dimensional space of the cavity polarization vector **ϵ** (eq 7) as follows
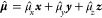
13and eq 12 can be further written as
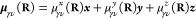
14where
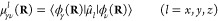
15Here μ_*γν*_^*l*^(**R**) are the permanent dipoles (γ = ν) and transition dipoles (γ ≠ ν) along different directions and are usually obtained from the *ab initio* electronic structure calculation for the realistic molecular system. Combining eq 7 and eq 14, the light–matter coupling term **ϵ**·**μ**_*γν*_(**R**) in eq 11 can be expressed as
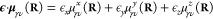
16

Besides the adiabatic-Fock state, one can further define the polaritonic state^3,42^ as the eigenstate of *Ĥ*_pl_ (see definition in eq 2) through the following eigenequation
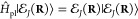
17where |(**R**)⟩ is the polariton state with polariton energy . The polariton eigenstate can be expressed as the linear combination of the adiabatic-Fock states
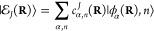
18where *c*_α,*n*_^*J*^(**R**) = ⟨ϕ_α_(**R**), *n*|(**R**)⟩ and (**R**) can be obtained by diagonalizing the matrix of *V̂* = *Ĥ*_pl_ (constructed from the adiabatic-Fock state basis in eqs 10 and 11) as
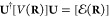
19where
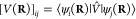
20with the basis |*ψ*_*j*_(**R**)⟩ defined in eq 10.

### 2.2 Quantum Dynamics Propagation

In this work, we use the TSH approach to propagate the quantum dynamics of the coupled electronic–photonic–nuclear DOFs. In particular, the electronic–photonic DOFs are treated as the quantum subsystem, whereas the nuclear DOFs are treated as the classical subsystem. To model the cavity loss (due to the imperfect cavity mirror that leads to a finite photon lifetime), we employ the Lindblad dynamics approach.^28,45,46^

The equation of motion (EOM) for the quantum subsystem (electronic–photonic DOF) is expressed as
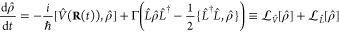
21where ρ̂ is the reduced density operator of the quantum subsystem (electronic–photonic DOFs), *V̂* = *Ĥ*_pl_ is the polariton Hamiltonian defined in eq 2, and *L̂* is a Lindblad jump operator^28^ that imparts the impact of the environment (photonic bath) onto the system (cavity mode) with interaction strength Γ (that has a unit of rate or inverse time) and {*Â*, *B̂*} = *ÂB̂* + *B̂Â* represents the anti-commutator. In eq 21, we further define two superoperators, *L*_*V̂*_[·] and *L*_*L̂*_[·], which are used to describe the evolution of reduced density governed by the quantum subsystem Hamiltonian *V̂* and jump operator *L̂*, respectively. The Lindblad jump approach ensures the total population conservation as well as the proper decoherence dynamics among states due to the population jumps.^28^ For the cavity loss process considered in this work, the jump operator *L̂* is defined as^28^
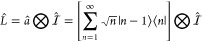
22where *â* is the photonic annihilation operator and *Î* is the identity in the electronic subspace. The above jump operator *L̂* only acts on the photonic DOFs with no impact on the electronic DOFs. The jump operator *L̂* = |0⟩⟨1| causes the population of state |1⟩ to decay with a rate of Γ, state |0⟩ to gain the population lost by state |1⟩, and state |1⟩ to decohere from every other state with a rate of Γ/2 (see detailed discussions around eq 24 of ref (28)). The Lindblad jump operator thus introduces the decoherence between photonic excitations and “photonic bath” DOFs, and the microscopic discussions can be found in Appendix D of ref (28).

Considering a short-time propagation from *t* to *t* + d*t* where d*t* is small, the time evolution of the density can be approximated as^28^
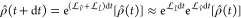
23Under the above approximation, we can propagate the density governed by the quantum subsystem Hamiltonian *V̂* and jump operator *L̂* separately, with EOMs as
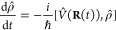
24a
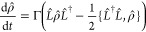
24bWith the adiabatic-Fock state defined in eq 10, the total wave function of the quantum subsystem is expanded as
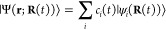
25where *c*_*i*_(*t*) is the expansion coefficient. The reduced density matrix element in the adiabatic-Fock state basis can be expressed as follows
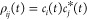
26Further, the quantum subsystem evolution equations (eq 24a and eq 24b) are also expanded in this adiabatic-Fock state basis as follows
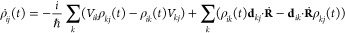
27a
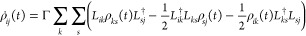
27bIn the above equations, **d**_*ij*_ is the non-adiabatic coupling (NAC) defined as
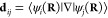
28We can write the matrix elements of **d** in the adiabatic-Fock state basis as
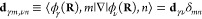
29because the Fock states do not explicitly depend upon **R** and are orthonormal to each other and **d**_*γν*_ is the regular NAC among the adiabatic electronic states of the molecule. Further, **Ṙ** is the velocity of the nuclear DOF. The matrix element of the jump operator *L̂* can also be written in the adiabatic-Fock state basis as
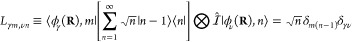
30Note that only the propagation of the quantum subsystem is presented above, while the nuclear DOFs are treated classically and propagated using the TSH method; see details in section 2.3.

### 2.3 Trajectory Surface Hopping

In this work, we use the TSH^47,48^ method to perform the on-the-fly non-adiabatic dynamics for the realistic molecular system coupled to the cavity. Here, we briefly describe the TSH dynamics for the coupled molecule–cavity hybrid system, whereas the details can be found in our previous work.^25^

We use the fourth-order Runge–Kutta method to integrate the propagation of the quantum subsystem with the Lindblad dynamics, through the EOMs presented in eq 27a and eq 27b. Specifically, for each nuclear time step, we propagate the quantum subsystem using eq 27a and then propagate using eq 27b. The classical subsystem (nuclear DOF) is propagated using Newton’s EOM with the velocity Verlet algorithm. In the TSH dynamics,^47^ the nuclear force comes from *only one* specific polariton state |(**R**(*t*))⟩ (eigenstate of *V̂*, see eq 17) as follows
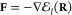
31where (**R**) is the energy of the *active* adiabatic polariton state and *I* is the active state index determined with the TSH algorithm, which will be determined at every nuclear propagation step. The nuclear gradient is calculated as
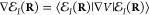
32as the results of the Hellman–Feynman theorem. Assuming the completeness relation ∑_*i*_ |*ψ*_*i*_⟩⟨*ψ*_*i*_| = *Î* (where |*ψ*_*i*_⟩ = |*ϕ*_*α*_(**R**), *n*⟩) and inserting it into eq 32, we have
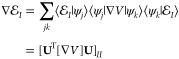
33where the transformation matrix **U** can be obtained through eq 19. The matrix element of ∇*V* is expressed as^25^
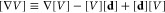
34where [*V*] and [**d**] are the matrix of *V̂* and derivative coupling operator in the adiabatic-Fock state basis and can be calculated with eq 11 and eq 29, respectively. The detailed derivation of the expression of [∇*V*] (eq 34) can be found in ref (25), based on the energy conservation condition of the mixed quantum-classical system (see eq 21 and eq 22 of ref (25)). As such, the gradient expressions in eq 31 and eq 34 rigorously conserve the total energy for the mixed quantum-classical system. We have also carefully tested the energy conservation for the polariton system when the cavity loss is not included (see Figure S9 in the Supporting Information).

To evaluate ∇[*V*], one needs to take the derivative on each term of *V* expressed in eq 11, including ∇*E*_*ν*_(**R**) and ∇(**ϵ**·**μ**_*γν*_(*R*)). The first term is the gradient of the adiabatic electronic state energy, which can be obtained from the *ab initio* electronic structure calculation. The second term can be further expanded using eq 16 as
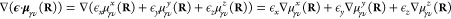
35where one needs to calculate the derivatives of the dipole matrix elements ∇μ_*γν*_^*l*^(**R**) for *l* = *x*, *y*, *z*. Unfortunately, these derivatives are not implemented for most of the electronic structure methods. We address this theoretical challenge using a machine learning model based on the Kernel ridge regression (KRR) method in this work, which will be extensively discussed in section 2.4.

To further obtain the switching probability of the molecular system from one polariton state to another polariton state, we follow the recently developed global flux surface hopping (GFSH) algorithm.^49^ This algorithm is shown to outperform the original fewest switches algorithm^47^ for systems with more than two electronic states. Here, we briefly describe how to apply it to polaritonic systems. First, we express the density matrix element in the *polariton basis* as follows
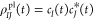
36where *c*_*I*_(*t*) is the expansion coefficient of the total wave function of the quantum subsystem in the polaritonic basis as
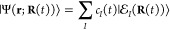
37For clarity, we denote the reduced density matrix in the adiabatic-Fock basis as ρ_*ij*_^af^(*t*), and ρ_*IJ*_^pl^(*t*) is the reduced density matrix in the polariton basis (expressed in eq 36). To obtain the density matrix in the polaritonic basis during the dynamics, we use the following unitary transformation
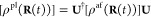
38We employ the GFSH algorithm^49^ to calculate the probability of switching from the active polariton state |⟩ to *any other* polariton state |⟩ during the time interval between *t* and *t* + *δt* as follows
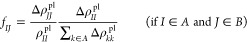
39where Δρ_*II*_^pl^ = ρ_*II*_^pl^(*t* + *δt*) – ρ_*II*_^pl^(*t*). From time *t* to *t* + *δt*, all the polariton states that lose population form group *A*, while all the polariton states that gain population form group *B*. Here we only need to calculate the switching probability when the current active state |⟩ belongs to *A*, and the destination state belongs to *B*. All other types of state switches, for example, |⟩ and |⟩ belong to the same subgroup or |⟩ belongs to *B* and |⟩ belongs to *A*, are not allowed, and the switching probabilities are set to 0 based on the algorithm.^49^ The non-adiabatic transition, i.e., stochastic switches from the currently occupied state |⟩ to another state |⟩, occurs if the following condition is satisfied
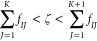
40where ζ is a uniform randomly generated number between 0 and 1 at each nuclear time step. If the transition is accepted, the active state is set to the new adiabatic state |⟩. The GFSH scheme is a natural generalization^49^ of Tully’s fewest switches surface hopping (FSSH) algorithm^47^ and has been shown to produce more accurate population dynamics for systems with more than two states. In addition, the hopping criteria of GFSH (eq 39) only require the knowledge of the population and the difference of population between two dynamics steps. This allows us to compute the hopping probability when propagating the quantum dynamics with the Lindblad equation (eq 27b), as discussed in the early work that combines the GFSH and Lindblad dynamics.^50^

For each nuclear time step, the density of the quantum subsystem is propagated using eq 27a and eq 27b. We have to calculate the hopping probabilities and assess if the system should hop to another state for both of these two steps. If a hopping event happens from current state |⟩ to new |⟩ due to quantum subsystem evolution itself governed by eq 27a, the velocities of the nuclei are rescaled along the direction of the NAC **d**_*IK*_(**R**) in order to conserve the total energy.^48^ In particular, the NAC between two polaritonic states can be expressed as^48^
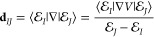
41Note that this should not be confused with the molecular derivative coupling **d**_*ij*_ defined in eq 28. One can further express eq 41 by inserting the completeness relation as
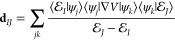
42This NAC in the polariton representation (eq 42) is only used for computing the direction of rescaling the velocity. Further, since the energy of the entire hybrid system is dissipated to the photonic environment during the cavity loss process, there is no energy conservation for the cavity loss process, and as such, we do not perform the velocity scaling for nuclear DOFs if the hopping occurs during the cavity loss process governed by eq 27b. This is consistent with the previous work of TSH dynamics in simulating polariton dynamics.^13,15,27^

In the TSH simulation used in this work, we set one of the polariton states as the initial active state, which means the initial coefficient *c*_*I*_(0) for the state |⟩ is set to be one, and the rest of the coefficients are set to be zero. These coefficients {*c*_*J*_(0)} in the polariton state basis can be unitary-transformed into the coefficients {*c*_*i*_(0)} in the adiabatic-Fock state basis to perform the Lindblad dynamics for each nuclear initial condition.

When computing the population dynamics in a representation that is *not* the adiabatic states of *V̂* = *Ĥ*_pl_, there is no unique way to calculate them in the TSH approach.^51^ In this work, we follow the estimator proposed by Subotnik et al.,^51^ which shows more accurate results in our previous work for polariton dynamics.^26^ Below, we briefly introduce this estimator.

To get the adiabatic-Fock state population of the |*ψ*_*i*_⟩ state ρ_*ii*_^af^ from the TSH simulation, the most straightforward way is through following unitary transformation
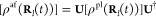
43where [ρ^pl^**R**_*l*_(*t*))] is the reduced density matrix in the polariton basis along a given nuclear trajectory **R**_*l*_(*t*), with *l* as the label of the trajectory. Further, **U**(**R**_*l*_(*t*)) is the matrix that diagonalizes the matrix [*V*(**R**_*l*_(*t*))] as shown in eq 19, along the same trajectory **R**_*l*_(*t*). The adiabatic-Fock state population is then obtained from trajectory average as follows
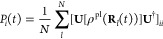
44where *N* is the total number of trajectories. Instead of calculating the polaritonic state density matrix [ρ^pl^(**R**_*l*_(*t*))] using eq 36 directly, in the estimator used in this work, we calculate the diagonal elements of ρ^pl^ using the active state index and calculate the off-diagonal elements using the polaritonic state expansion coefficients {*c*_*I*_(*t*)}
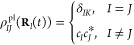
45where *K* is the active polaritonic state. This estimator was developed in connection with the mixed quantum-classical Liouville equation^51^ and has been shown to provide a more accurate diabatic population,^51^ as well as adiabatic-Fock states populations for a Shin–Metiu model coupled to the cavity.^26^ For the population dynamics of the polariton states |(**R**)⟩, we use the traditional active estimator, which is
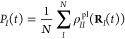
46We have also reported the *trans* isomer population, computed as
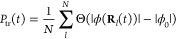
47where Θ is the Heaviside function, |ϕ(**R**_*l*_(*t*))| is the absolute value of the CNNC dihedral angle along the **R**_*l*_ trajectory, and |ϕ_0_| = 90° is the dividing surface that distinguishes the *cis* and *trans* isomers. The details of polariton dynamics with the TSH method can also be found in our previous work performing QED dynamics with the MQC methods.^25,26^

### 2.4 Kernel Ridge Regression Model for Dipoles

As pointed out in section 2.3, the dipoles, including the permanent dipoles and transition dipoles, and their derivatives are necessary ingredients to perform polariton dynamics simulations. However, for realistic molecular systems, the derivatives of dipoles are not readily available for most of the commonly used excited-state electronic structure methods, such as CASSCF and TD-DFT. Therefore, we circumvent this technical difficulty by employing the *machine learning* techniques in this work to get the *analytical expressions* of dipoles in terms of the molecular geometry μ(**R**) = *f*(**R**). After that, we have full access to the analytical expression of derivatives of dipoles using ∇μ(**R**) = ∇*f*(**R**). Below, we will briefly discuss the machine learning strategy we used to parametrize dipoles, and the details can be found in the previous work on performing on-the-fly non-adiabatic dynamics with the machine-learned potential energy surfaces (PESs).^52^

To obtain the relation between the dipoles and the molecular geometry, we first need to define a proper molecular descriptor to represent the molecular geometry. One simple molecular descriptor is the Coulomb matrix^53^**M**, with the matrix elements defined as follows
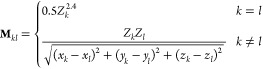
48where *Z*_*k*_ is the atomic number of atom *k* and (*x*_*k*_, *y*_*k*_, *z*_*k*_) are the Cartesian coordinates of atom *k*. Note that the diagonal elements of the Coulomb matrix are not dependent on molecular configuration, and thus, it is safe to remove them in the construction of the molecular descriptor. In addition, the Coulomb matrix is symmetric; thus, we only need to consider the off-diagonal elements of the upper triangle part. All of these used off-diagonal elements define a vector ***m*** that contains *N*_*a*_ × (*N*_*a*_ – 1)/2 elements (where *N*_*a*_ is the number of atoms of a single molecule) and are used as input in the training and prediction process. The Coulomb matrix provides a simple and effective representation of molecular geometry, which takes both element types and internal distances into account and has shown great advantages in building machine learning models as demonstrated in the previous work.^31,52−55^

In this work, we employ the KRR method in the machine learning process. KRR is one of the most popular *supervised* learning approaches and has been used for the prediction of molecular properties in several studies.^52,53,56^ In the KRR approach, the molecular property *f*(***m***), such as the molecular dipole considered in this work, is estimated by a function of the nuclear configurations represented by the molecular descriptor ***m***. The model assumes that the property can be calculated as follows
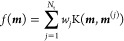
49where ***m***^(*j*)^ is the molecular descriptor for the *j*th configuration, *N*_t_ is the number of configurations in the training data set, and *w*_*j*_ is the regression coefficient. Further, K is the kernel function. In this work, we use the radial basis function (RBF) kernel in the learning algorithms. The RBF kernel is often called the Gaussian kernel, defined as follows
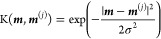
50where σ is the kernel width and ***m*** – ***m***^(*j*)^ is the Euclidean distance between the two molecular descriptor vectors ***m*** and ***m***^(*j*)^. The regression coefficients *w*_*j*_ are trained by minimizing the following expression
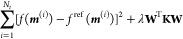
51where *f*^ref^ is the reference molecular property value. In eq 51, the first term is typically referred to as the L2-norm (also known as the Euclidean norm or the squared magnitude) of the model parameters, which corresponds to the sum of the squares of the weights assigned to each feature in the model. This is used for all linear regression models. The KRR model requires a second penalty term in eq 51, related to the kernel matrix **K**, the coefficient matrix **W**^T^ = (*w*_1_, ..., *w*_*j*_, ..., *w*_*N*_t__), and λ is a regularization parameter used to prevent overfitting. By adding the second penalty term, the KRR model is encouraged to have smaller parameter values, which in turn makes the model less sensitive to noise in the training data and less likely to overfit. The amount of regularization (i.e., the strength of the penalty term) is controlled by a hyperparameter (λ in eq 51), which needs to be tuned using cross-validation or other techniques to find the optimal value for the given problem. Overall, the penalty term is an important component of KRR that helps to balance the trade-off between fitting the training data well and having good generalization performance on unseen data.

The kernel matrix **K** is obtained by eq 50 over all pairwise distances between all training data, After we obtain the regression coefficients *w*_*j*_, the molecular property *f*(***m***) is calculated using eq 49.

The first-order derivative of *f*(***m***) with respect to Cartesian coordinates (*x*_*k*_, *y*_*k*_, *z*_*k*_) of atom *k* can be then calculated by the chain rule
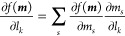
52where *l* = *x*, *y*, *z* and *m*_*s*_ is an element of ***m***.

During the process of the training and prediction of the dipoles and their derivatives, the Coulomb matrix is used as the molecular descriptor, which is invariant to the translational and rotational motion of molecules. However, the dipoles of the molecular system are vectors and will change if we rotate the molecule. Therefore, we define a relative Cartesian coordinate system in this work, which depends on the relative positions between the atoms of the molecule. The dipoles used in the training and prediction process are based on the relative Cartesian coordinate system, which will also be invariant to the translational and rotational motion of molecules. The details of this implementation can be found in the Supporting Information.

## 3 Computational Details

Here, we briefly discuss the computational details, related to the electronic structure calculation, the training process of the ML model, and details of the non-adiabatic polariton dynamics simulations. The source code for the polariton surface hopping dynamics, the source code for the machine learning, and an example of training the dipole moment are provided in a GitHub repository.^57^

### 3.1 Electronic Structure Calculation

The ground-state minima of the two isomers (*cis* and *trans*) of the azomethane molecule were optimized using the DFT method with the B3LYP functional. The frequency analysis was performed to confirm these minima are stationary. The vibrational frequencies were further used in the Wigner sampling process. The CASSCF method^58,59^ was used to obtain the adiabatic energies, gradients, dipoles (permanent and transition dipoles), and NAC of the azomethane molecule in the electronic structure calculations. Following the previous studies,^39−41^ a two-state average with an active space of six electrons in four orbitals [SA-2-CAS(6,4)] was used in the CASSCF calculations. In all electronic structure calculations, the 6-31G* basis set was used. The DFT and CASSCF calculations were performed using the Gaussian 16^60^ and Molpro 2015^61^ packages, respectively.

**Figure 1:**
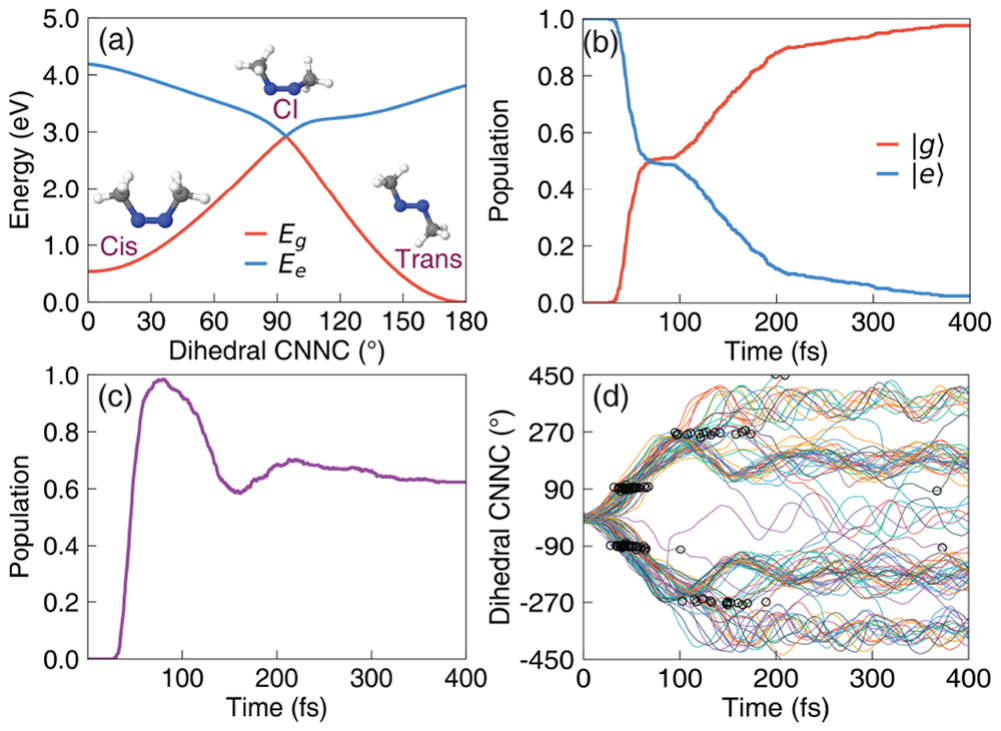
Non-adiabatic dynamics of the azomethane molecule outside the cavity. (a) The potential energy surface of the |*g*⟩ and |*e*⟩ states along the reaction pathway (obtained using linear interpolation of the internal coordinates) from the *cis* isomer to the conical intersection configuration and to the *trans* isomer for the azomethane molecule; (b) the electronic population of the |*g*⟩ and |*e*⟩ states; (c) the population dynamics of the *trans* isomer; (d) time-dependent value of the dihedral CNNC angle (of 100 trajectories), with the black circles indicating where the surface hopping events happen from the |*e*⟩ state to the |*g*⟩ state during the trajectory surface hopping simulations.

**Figure 2:**
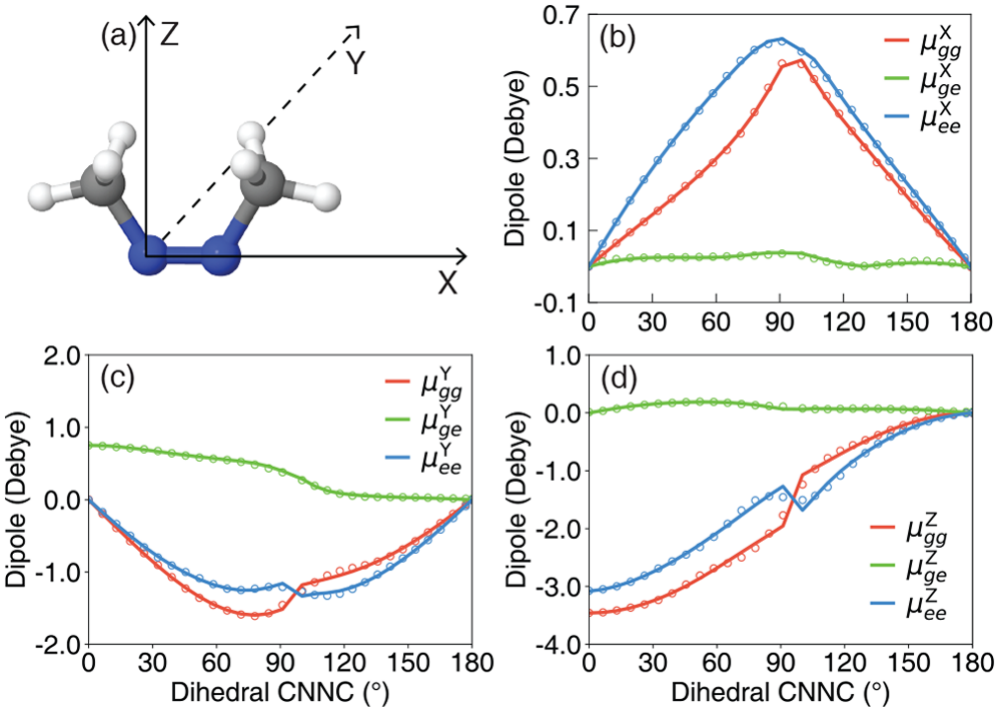
(a) The definition of the global reference Cartesian coordinate system used in this work. The optimized *cis* isomer is used as the reference, where the *X*-axis is defined along the NN double bond, the *Y*-axis is defined perpendicular to the molecular plane, and the *Z*-axis is defined perpendicular to the *X*- and *Y*-axes simultaneously. These three directions are also used to define cavity field polarization directions. (b–d) The permanent and transition dipole components along the reaction pathway from the *cis* isomer to the conical intersection configuration and to the *trans* isomer, projected along the (b) *X* direction, (c) *Y* direction, and (d) *Z* direction. Results are obtained from the KRR (open circles) and *ab initio* CASSCF calculations (solid lines).

**Figure 3:**
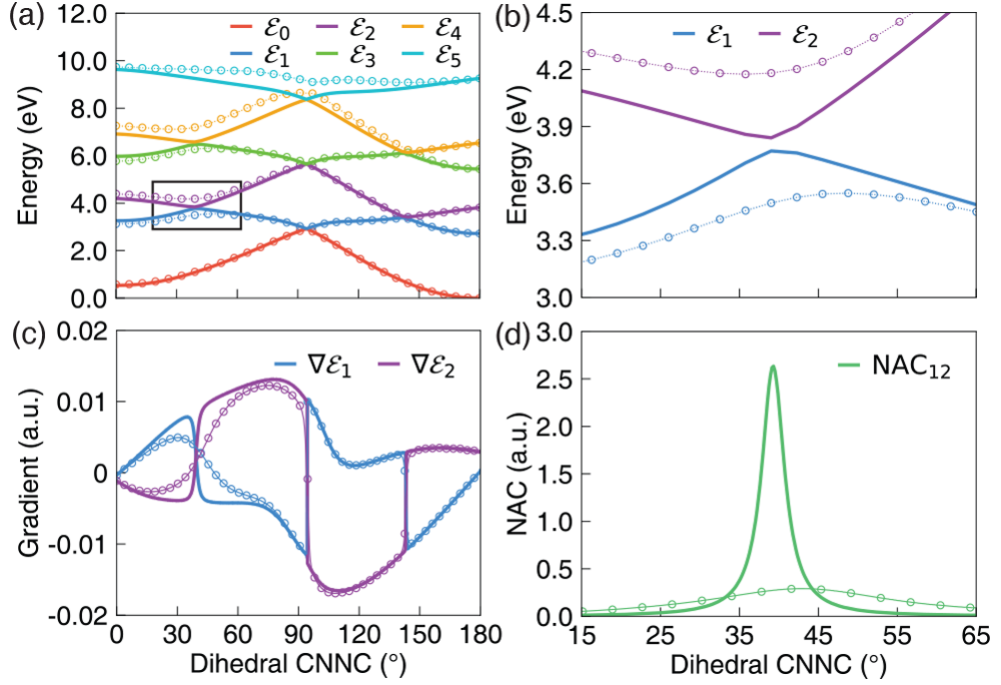
Polariton potentials, gradients, and non-adiabatic couplings along the reaction pathway from the *cis* isomer to the conical intersection configuration and to the *trans* isomer for the molecule–cavity hybrid systems. The cavity frequency is set to ℏω_c_ = 2.72 eV. The field is polarized along the *Y*-axis. The results were obtained with light–matter coupling strengths of *g*_c_ = 0.005 au (solid line) and *g*_c_ = 0.05 au (open circles). (a) Polariton potential energy surface (*R*). (b) The zoomed-in plot of the polariton potential of the |⟩ and |⟩ surfaces. (c) Nuclear gradients associated with the |⟩ and |⟩ states. (d) Non-adiabatic coupling ⟨|∇|⟩ between the two polariton states.

It is reported that the conical intersection (CI) between the |*g*⟩ and |*e*⟩ states plays an important role in the photoisomerization process of the azomethane molecule.^39,40^ Thus, we optimized the molecular geometry of the CI between the ground state and the first excited state (see the inset geometry with “CI” label in Figure 1a) using the geometry optimization method proposed by Yarkony and co-worker,^62^ as implemented in the Molpro 2015^61^ package. The CI-optimized geometry is obtained at the SA-2-CAS(6,4) level of theory. We also obtained the optimized geometries of both *cis* and *trans* isomers in the ground electronic state, at the level of DFT using B3LYP/6-31G*. The entire isomerization reaction path was generated by linear interpolation in internal coordinates from the CI geometry to both the *cis* isomer and the *trans* isomer. We generated a total of 60 of the geometries along the reaction path, and they are not further optimized (as such, they are just a rigid interpolation away from the CI point to the *cis* and *trans* isomers). These geometries are used to plot the PESs in Figure 1a and for visualizing the molecular dipoles in Figure 2 and the polariton PESs and gradients in Figure 3.

### 3.2 Training Procedure of the Machine Learning Model

In this work, we employed the KRR method to train the analytical expression of dipoles (permanent and transition dipoles) for the azomethane molecule. All of the training data are generated at the SA-2-CAS(6,4) level of theory, with the 6-31G* basis set. This ensures the dipole and its derivative of it are also at the level of CASSCF, which will be consistent with the other nuclear gradient (see eq 34) used in the dynamics simulations. Below, we briefly describe our training procedure.

First, we generated a number of initial conditions (nuclear coordinates and momenta) near the ground-state minimum of the *cis* isomer using the Wigner sampling at *T* = 0 K. Based on these initial conditions, we performed the on-the-fly Born–Oppenheimer molecular dynamics (BOMD) simulations at the |*g*⟩ and |*e*⟩ surfaces with 100 and 200 trajectories, respectively. The nuclear motions are propagated using the velocity-Verlet algorithm with a time step of d*t* = 0.5 fs. A total of 30 nuclear steps were run for the dynamics at the |*g*⟩ state, and 700 nuclear steps were run for the dynamics at the |*e*⟩ state. To enhance the sampling of the distribution of the geometries in the dynamics process, we further generate another 200 initial conditions using the Wigner sampling at *T* = 1000 K and performed the BOMD simulations at the |*e*⟩ state. A total of 100 nuclear propagation steps are run for each trajectory. We performed the same initial sampling and dynamics for the *trans* isomer. In addition, we created 200 conformations by rotating the central NN double bond from the *cis* isomer to the *trans* isomer and performed short-time (30 nuclear steps) BOMD at the |*e*⟩ state initialized from these conformations.

Second, we collected snapshots of every two nuclear steps (with a time separation of 1 fs) in all the above dynamics to form our data set library, with a total of 166,000 azomethane conformations). We randomly chose 120,000 azomethane conformations to build the preliminary training data set and chose the rest of the 46,000 configurations to build the testing data set. As one can see, the training data set has a huge number of data points. On the one hand, if we used all of these data in the KRR regression, the fitting procedure may become extremely slow and the regression process would require a huge amount of computer memory. On the other hand, in the sampling process, several data points may be located within the same areas, while much fewer points in some other areas. Thus, it is useful to create a balanced description of the data distribution over all important spaces in the regression, as suggested by previous work.^52,54^ The most direct way is to perform the *clustering analysis* of data points before training.

In this work, we took the similarity between the Coulomb matrix of different geometries as the reference to perform clustering with the hierarchical agglomerative clustering algorithm.^63^ The training data set was clustered into 1000 groups. Since the data is not well distributed, when we cluster them into subgroups, the number of data points in each subgroup varies. The number of data points in some subgroups is less than 100, and in this case, we selected all the data (geometries) in these subgroups. Otherwise, we selected at most 100 geometries from each group. All selected geometries formed the final training data set, which contains a total of 72,993 azomethane conformations. The wide distribution of the CNNC dihedral angle (see Figure S2 in the Supporting Information), which is the main reaction coordinate during the isomerization process, indicates that the training data set is properly sampled in this work.

Finally, we performed the KRR method to fit the machine learning dipoles based on the final training data set and then we tested their prediction ability based on the testing data set. The parameters σ and λ in the training process were set to 0.05 and 0.005 to get the smallest training and test errors. After obtaining the machine-learned dipoles, we get their derivatives using eq 52. All the machine learning relevant codes, such as KRR, data prescreening, and the interface with dynamics, were written with the Python language, based on the scikit-learn toolkit.^64^

### 3.3 Non-adiabatic Polariton Dynamics Simulations

The GFSH approach^49^ was employed in the non-adiabatic dynamics simulations both inside and outside the cavity in this work. The initial condition is generated from a separate (independent) set of initial nuclear configurations near the ground-state minimum of the *cis* isomer using the Wigner sampling at *T* = 0 K. The initial Wigner distribution is sampled from the ground vibrational state ν = 0 on the ground electronic state |*g*⟩, where the normal-mode frequencies (in the harmonic approximation) are calculated based on the approach outlined in refs (65 and 66), as implemented in the JADE-NAMD package.^67^ In particular, the nuclear density ρ_W_(**R̃**, **P̃**) in terms of the molecular normal-mode frequencies {ω̃_*k*_} and phase space variables {**R̃**, **P̃**} is given as^68^
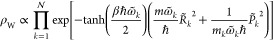
53The initial distribution {**R**, **P**} is then obtained by transforming {**R̃**, **P̃**} from the normal mode representation to the primitive coordinates using the unitary transformation that diagonalizes the Hessian matrix. All of the initial nuclear geometries for the quantum dynamics simulations are then sampled from the geometries centered at the optimized *cis* isomer (Figure 2a), which is also used as the reference configuration to define the global Cartesian system used in this work; see section 4.2 for details. The detailed geometry for each trajectory, of course, fluctuates around the *cis* isomer, governed by the Wigner distribution. As the polariton dynamics proceed, the molecule will move and rotate in space. We do not remove any center of mass translation and rotation during the dynamics simulations. These motions will change the instantaneous light–matter coupling strength due to the way the dipole vector orients relative to the field polarization direction, as well as the corresponding nuclear gradients.

The trajectories started from the |*e*⟩ state for dynamics outside the cavity and from the third polaritonic state |⟩ for the polariton dynamics when coupling the molecule inside the cavity. For all dynamics, the nuclear motion was propagated using the velocity-Verlet algorithm with a time step of d*t* = 0.2 fs. In each nuclear time step, 100 steps are used for the quantum subsystem propagation using eq 27a and eq 27b. All of the energy, gradient, and derivative couplings of the molecules are computed on the fly. All of the population dynamics results are obtained by averaging over 500 trajectories, using the expression of eq 44 (for adiabatic-Fock states) and eq 46 (for polariton states). The decoherence correction algorithm proposed by Granucci et al.^69^ was employed in the GFSH dynamics, and the decoherence parameter α was set to 0.1. This decoherence correction is performed for each nuclear time step. Note that this decoherence scheme provides a correction to over-coherence between electronic and nuclear degrees of freedom, whereas the Lindblad jump operator in eq 22 accounts for the decoherence between the cavity mode and “photonic bath” degrees of freedom due to the cavity loss mechanism. Both sources of decoherence (caused by electron–nuclear interactions and cavity–photonic bath interactions) are physical and need to be accounted for separately.

Below, we briefly summarize the details of performing the polariton dynamics. First, we use eq 53 to generate the initial nuclear condition, including the initial positions and momenta. Then, we use eq 27a and eq 27b to propagate the quantum dynamics of the electronic–photonic DOFs, while the nuclear DOFs are propagated with force from the active polaritonic state calculated using eq 33. In the construction of the system’s Hamiltonian and calculation of the nuclear forces, we use the machine learning model to get the dipoles and their derivative using eq 49 and eq 52, respectively, whereas all of the other quantities, including all energy, gradient, and derivative couplings, are computed on-the-fly using CASSCF. During the TSH dynamics, we use eq 39 to calculate the hopping probability from a current active state to any other states and use the Monte Carlo algorithm presented in eq 40 to stochastically determine to which state the system will hop. Finally, we use eq 44 and eq 46 to obtain the population of the adiabatic-Fock state and polaritonic state, respectively. All the dynamics calculations were performed with a development version of the JADE-NAMD package.^40,52,67,70,71^

## 4 Results and Discussion

### 4.1 Non-adiabatic Dynamics of the Molecule Outside the Cavity

Figure 1a presents the electronic adiabatic PESs for |*g*⟩ (red curve) and |*e*⟩ (blue curve) along the reaction path, with geometries obtained using the procedure described in section 3.1. The inset depicts the molecular structures of azomethane. Two isomers, *cis* and *trans*, exist for this molecule, defined by the CNNC dihedral angle at ∼0° and ∼180°, respectively. These two isomers can convert to each other upon photoexcitation.^72^ To explore how the photoisomerization process of azomethane can be influenced by the light–matter coupling, we first perform the non-adiabatic dynamics of azomethane *outside* the cavity. Although the *trans* isomer is energetically more stable (with a lower energy of ∼0.5 eV) than the *cis* isomer in the ground state (|*g*⟩), we start the dynamics from the *cis* isomer upon a Franck–Condon excitation, due to a larger transition dipole between the ground state (|*g*⟩) and the first excited state (|*e*⟩) at the *cis* configuration compared to that of the *trans* isomer.

Figure 1b presents the time-dependent adiabatic population in the TSH dynamics process. After being excited to the |*e*⟩ state, the system decays fast and nearly half of the trajectories hop to the |*g*⟩ state within ∼50 fs. The fast excited-state population decay process can be explained by the shape of the PES of the |*e*⟩ state (see Figure 1a), where no energy barrier exists along the reaction pathway from the *cis* isomer to the CI. After 50 fs, the decay of the |*e*⟩ state becomes slower and almost all the trajectories are at the |*g*⟩ state after ∼330 fs.

Figure 1c presents the time-dependent population of the *trans* configuration during the photoexcitation dynamics. In the beginning, all trajectories have the configurations of the *cis* isomer, and thus, the *trans* population is zero. This is correlated with the CNNC dihedral angles presented in Figure 1d, where all trajectories are ∼0° at *t* = 0. Then the system starts to rotate through the central NN bond and isomerize, with the CNNC dihedral angle passing by 90° or −90°, and the population of the *trans* configuration almost increases to 1 at ∼70 fs. Many trajectories hop to the ground state in the vicinity of CI, where the CNNC dihedral angles are ∼90° or ∼ –90°, as shown in Figure 1d. The trajectories hop to the ground state and then get trapped in the potential minimum of the *trans* isomer configuration (see Figure 1a). These trajectories will oscillate around 180° or −180° for the long-time dynamics. Other trajectories will keep rotating and go back to the *cis* configuration with the CNNC dihedral angles passing by 270° or −270°. Similarly, they are also trapped in the potential minimum of the *cis* isomer and oscillate around 360° or −360°. The rotation from the *trans* isomer back to the *cis* isomer of some trajectories explains why the population in Figure 1c decreases after ∼70 fs. The population of the *trans* configuration does not change too much after ∼300 fs, where most of the trajectories are already at the |*g*⟩ state, as shown in Figure 1b.

### 4.2 Machine Learning Model for Dipoles

To perform the non-adiabatic QED simulation with the TSH method, one needs to obtain the molecular dipoles and their derivatives with respect to the nuclear coordinates (see eq 11 and eq 35). In this work, we use the KRR method to obtain a machine learning expression of the analytical expression of the dipoles. We then obtain their derivatives using eq 52 to compute the contribution of the nuclear gradient due to the derivative of dipoles. As we know, the dipoles of the molecular system, including the permanent dipole and transition dipole, are vectors and have three components in space. Figure 2a presents the global Cartesian coordinate system defined in this work, which is used to determine the components of dipoles in different directions. For convenience, the optimized geometry of the *cis* isomer is used as the reference geometry (with the *XYZ* coordinates provided in the Supporting Information). As illustrated in Figure 2a, the *X*-axis is defined along the NN bond, the *Y*-axis is defined as the direction that is perpendicular to the molecular plane (the plane of the CNNC bond of the cis isomer), and the *Z*-axis is defined perpendicular to the *X*- and *Y*-axes simultaneously. Note that the field polarization direction (used in eq 7) is also defined in the axis depicted in Figure 2a. We emphasize that the global Cartesian coordinate system defined in Figure 2a is a reference coordinate used in the polariton dynamics process and is different from the relative Cartesian coordinate system, which is only used in the machine learning processes; see the Supporting Information for details.

Figure 2b–d presents the dipoles along the reaction pathway from the *cis* isomer to the *trans* isomer, obtained with the KRR method (open circles) and the CASSCF calculations (solid lines). As one can see, the gradients of these dipoles (slope of the curves in Figure 2b–d) could get very large, depending on the nuclear configurations, and it is often a drastic approximation to ignore those gradient components in the polariton non-adiabatic simulations. We can see that the machine-learned dipoles are consistent with the CASSCF results in all directions. We have also performed additional tests to check the accuracy of our machine-learned dipoles. Figure S3 in the Supporting Information shows the distribution of the test errors in the learning procedure for the machine-learned dipoles. Note that there are small discrepancies between our machine-learned model and the actual CASSCF data around the CI configuration (with a CNNC angle of ∼94°), where the characters of the |*g*⟩ and |*e*⟩ states exchange with each other, along with the exchange of their permanent dipoles. There is a “sudden” flip of the permanent dipoles of the |*g*⟩ and |*e*⟩ states before and after the CI, which is indicated in the CASSCF-calculated results. However, since the machine-learned dipoles are continuous functions of the nuclear coordinates (eq 49), the change of the permanent dipoles obtained with the KRR for each adiabatic electronic state becomes “smooth” around the CI. Considering that the NAC between the |*g*⟩ and |*e*⟩ states is very large near the CI region and plays a major role when the system arrives at that region in the dynamics process, the discrepancies between the machine-learned dipoles and the CASSCF dipoles near the CI region will not significantly impact the dynamics inside the cavity, which will be discussed in the next sections.

As shown in Figure 2b–d, the transition dipoles of the *trans* isomer (with the CNNC dihedral angle being ∼180°) are zero in all directions due to the symmetry of the *trans* isomer, while the transition dipole of the *cis* isomer has a finite value (∼0.8 D) in the *Y* direction. Hence, the |*e*⟩ state is a *dark* state at the *trans* configuration, while it is a *bright* state at the *cis* configuration. This is also the reason why we chose the *cis* isomer as the initial configuration to perform the dynamics, as mentioned in section 4.1. In addition, we note that, near the Franck–Condon region, the permanent dipoles are much smaller than the transition dipole along the *Y*-axis. In contrast, the permanent dipoles are much larger than the transition dipole along the *Z*-axis. Moreover, compared to the dipoles along the *Y*- and *Z*-axes, the dipoles along the *X*-axis are much smaller (<0.7 D). As a result, we will only perform the ab initio molecular cavity QED simulations with the field polarized along the *Y*- and *Z*-axes in this work.

In the Supporting Information, we further provide the polariton PESs obtained from quantum optics models, including the commonly used Jaynes–Cummings model and quantum Rabi model. The accuracy and validity of commonly used model systems need to be carefully assessed before adapting them to the field of molecular cavity QED. Unfortunately, these well-established approximations in the atomic cavity QED can explicitly break down in the molecular cavity QED, as shown in Figures S4 and S5. Thus, one needs to use the most rigorous Hamiltonian to describe the light–matter interactions and try to avoid unnecessary approximations. Accordingly, one should use a rigorous QED Hamiltonian (such as eq 2) to describe the light–matter interactions and avoid unnecessary approximations.

### 4.3 Ab Initio On-the-Fly Polariton Quantum Dynamics

Next, we present the *ab initio* polariton dynamics simulation results obtained using the TSH method described in section 3.3. In this section, the field is polarized along the *Y*-axis, which means (ϵ_*x*_, ϵ_*y*_, ϵ_*z*_) in eq 16 is set to be (0, 1, 0). In this case, only the component of dipoles along the *Y*-axis will contribute to the polariton dynamics. Two different light–matter coupling strengths, *g*_c_ = 0.005 au and *g*_c_ = 0.05 au, are used here. These light–matter couplings cause the Rabi splitting between the polariton states |⟩ (commonly referred to as the upper polariton state) and |⟩ (commonly referred to as the lower polariton state), with ℏΩ_R_ = 68 meV (for *g*_c_ = 0.005 au) and ℏΩ_R_ = 680 meV (for *g*_c_ = 0.05 au). In our simulations, we will consider a range of cavity loss, with the largest one being Γ = 64 meV. Note that strong coupling in cavity QED refers to ℏΩ_R_ ≫ Γ (as well as larger than the molecular excitation decay rate). As such, *g*_c_ = 0.05 au always satisfies the strong coupling condition and, for *g*_c_ = 0.005 au, one needs to consider the cavity loss rate Γ.

Figure 3a presents the PESs of the polaritonic states (see eq 17) along the reaction pathway from the *cis* isomer to the *trans* isomer, with the cavity frequency ℏω_c_ = 2.72 eV. We use two adiabatic electronic states (|*g*⟩ and |*e*⟩) and three Fock states (|0⟩, |1⟩, and |2⟩) in the construction of the adiabatic-Fock state basis, which produces six polaritonic states, all of which are included in the dynamics process. The PESs are obtained with the light–matter coupling strength *g*_c_ = 0.005 au (depicted in solid lines) and *g*_c_ = 0.05 au (depicted in open circles). Figure 3b provides a zoom-up of the square region highlighted in Figure 3a. An avoided crossing exists between the polaritonic states |⟩ and |⟩ due to the light–matter coupling, and the energy gap (the so-called Rabi splitting) increases along with the increase of the coupling strength. Figure 3c presents the nuclear gradients at the |⟩ and |⟩ states, and Figure 3d presents the NAC between them along the reaction pathway. The one-dimension gradients and NAC for each geometry along the reaction pathway are obtained by projecting the gradients and NACs in the whole space (including all nuclear DOFs) to the vector defined by the difference between the Cartesian coordinates of geometries before and after the current geometry. The gradients are consistent with the change of the PESs (Figure 3a), and the NAC between the  and  states decreases when the light–matter coupling strength increases as expected.

**Figure 4:**
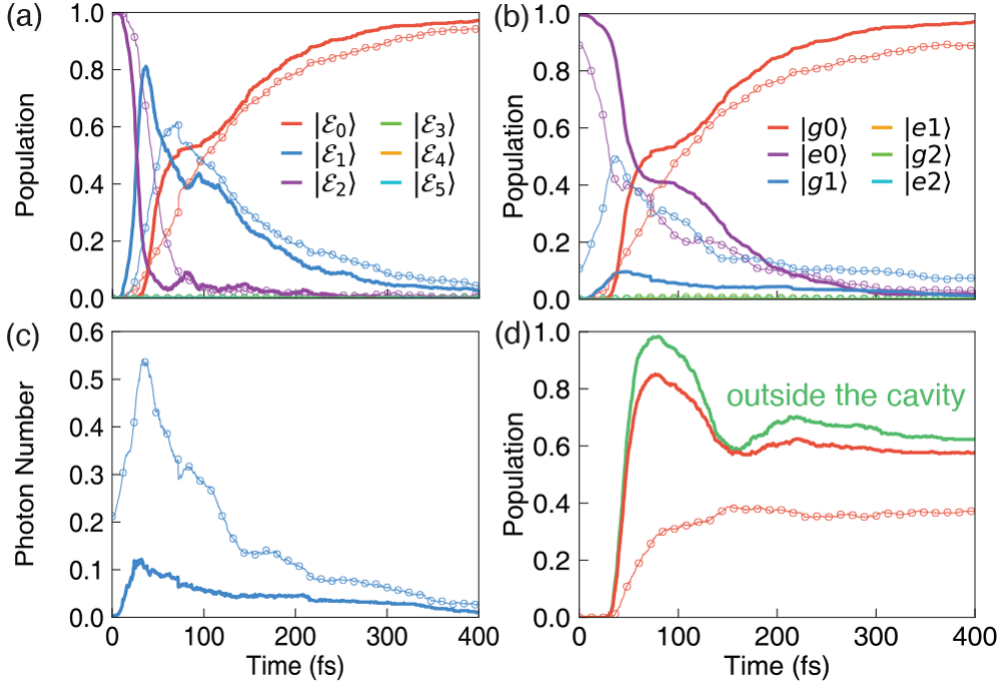
Time-dependent properties obtained from the *ab initio* on-the-fly molecular cavity QED simulations. The cavity loss rate is set to Γ = 4 meV. The field is polarized along the *Y*-axis. The cavity frequency is set to ℏω_c_ = 2.72 eV. The results obtained with light–matter coupling strengths of *g*_c_ = 0.005 au (solid lines) and *g*_c_ = 0.05 au (open circles). (a) Population of the polaritonic states (see eq 18); (b) population of the adiabatic-Fock states (see eq 10); (c) the average photon number population inside the cavity; (d) population of the *trans* isomer outside the cavity (green solid) and inside the cavity (red curves), with *g*_c_ = 0.005 au (red solid line) and *g*_c_ = 0.05 au (red open circles).

Figure 4 presents the results of our *ab initio* polariton quantum dynamics simulation, using the CASSCF *ab initio* calculation for all quantities, except for dipoles and their derivatives which are obtained from the machine learning model trained at the CASSCF level. Figure 4a presents the population dynamics of the polaritonic states, with the light–matter coupling strengths *g*_c_ = 0.005 au (depicted in solid lines) and *g*_c_ = 0.05 au (depicted in open circles). When the light–matter coupling is relatively small (with coupling strength *g*_c_ = 0.005 au), the initial polariton population in |⟩ decays very fast to the |⟩ state in the first 60 fs of the dynamics. The population of the |⟩ state begins to grow at ∼30 fs, and nearly all trajectories have hopped to the |⟩ state at the end of the dynamics. Other high-lying excited states, like |⟩, |⟩, and |⟩, are barely populated during the polariton dynamics process. Besides the polaritonic populations, we can also understand the population dynamics in the adiabatic-Fock states, as shown in Figure 4b. Since the light–matter coupling is weak when *g*_c_ = 0.005 au (depicted in solid lines), the |⟩ state near the Franck–Condon region is mostly composed of the |*e*0⟩ state. Thus, the population of the |*e*0⟩ state is nearly 1 at the beginning of the dynamics. Then, the population transfers to the |*g*0⟩ state due to the electronic NAC between |*e*⟩ and |*g*⟩ states. During this process, we can see a small population of the |*g*1⟩ state, which is induced by the light–matter coupling dominated by the transition dipole between |*e*⟩ and |*g*⟩.

When the light–matter coupling strength is increased to *g*_c_ = 0.05 au, the results are plotted with open circles in Figure 4. First, the transition process of the system from the |⟩ state to the |⟩ state slows down (Figure 4a) due to the decrease of the NAC between the |⟩ and |⟩ states (Figure 3d). The decay process of the system to the |⟩ is also postponed; see Figure 4a. Second, the population of the |*g*1⟩ state is largely increased due to the increase of the light–matter coupling strength, as shown in Figure 4b.

Figure 4c presents the number of photons during the dynamics process. Note that *â*^†^*â* is not the “photon number” operator under the dipole gauge used in the PF Hamiltonian^73,74^ because the rigorous photon number operator should be obtained by applying the Power–Zienau–Woolley (PZW) Gauge transformation^44,75,76^ on the photon number operator *â*^†^*â*. The correct photon number operator under the dipole gauge is
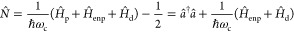
The time-dependent photonic population is then computed as ⟨Ψ(*t*)|*N̂*|Ψ(*t*)⟩ = Tr[ρ̂(*t*)*N̂*] = ∑_*ij*_* ρ*_*ij*_(*t*)⟨*ψ*_*j*_|*N̂*|*ψ*_*i*_⟩, with the polariton wave function |Ψ(*t*)⟩ expressed in eq 25 and the reduced density *ρ*_*ij*_(*t*) in eq 26. Since most of the photonic character is contributed from the |*g*1⟩ state, the evolution of the photon number is very similar to that of the population of the |*g*1⟩ state as shown in Figure 4b. For a larger light–matter coupling strength (*g*_c_ = 0.05 au), because of a larger population for the |*g*1⟩ state (Figure 4b), the resulting photon number inside the cavity is also larger.

Figure 4d presents the time-dependent population of the *trans* configurations when coupling the molecule to the cavity. In particular, we compare the case of the trans population dynamics outside the cavity (green solid line) with the case of coupling the molecule inside the cavity, with the coupling strength of *g*_c_ = 0.005 au (red solid lines) and *g*_c_ = 0.05 au (red open circles). We have observed a significant suppression of the trans population when the molecule is strongly coupled to the cavity. As such, this simulation demonstrates the suppression of the isomerization reaction from cis-to-trans configuration, when considering realistic cavity loss. The reason for the suppression of the cis-to-trans reaction is that more trajectories are staying on the |⟩ state after leaving the Franck–Condon region due to the *decrease* of the *polaritonic* NAC between the |⟩ state and |⟩ state, as clearly shown in Figure 3d. The above results clearly demonstrate that the chemical reaction of the molecular system can be controlled by the light–matter coupling, and even effectively controlled by tuning the light–matter coupling strength, agreeing with what we have observed in our previous work using a simple model system.^6^

**Figure 5:**
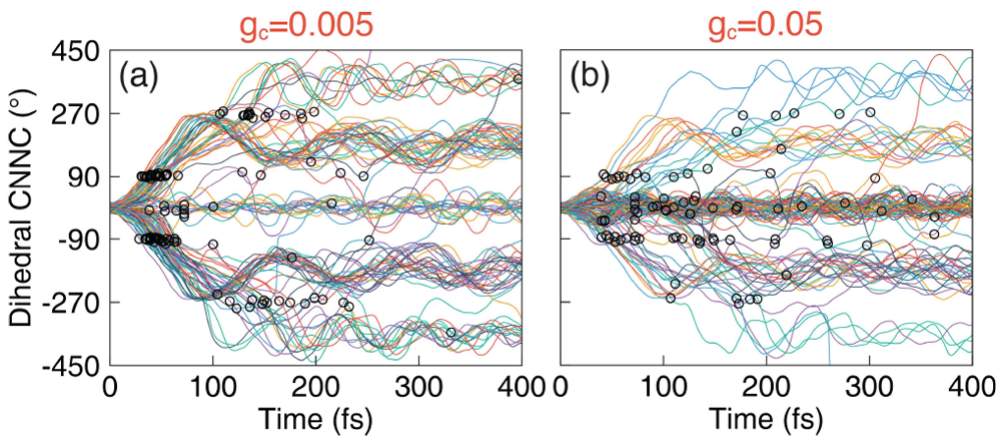
Time-dependent CNNC dihedral angles of 100 trajectories generated from the QED simulations of the azomethane molecule coupled to the cavity, with light–matter coupling strengths of (a) *g*_c_ = 0.005 au and (b) *g*_c_ = 0.05 au. The cavity loss rate is set to Γ = 4 meV. The field is polarized along the *Y*-axis. The cavity frequency is set to ℏω_c_ = 2.72 eV. The black circles indicate where the surface hopping events happen from the |⟩ state to the |⟩ state during the trajectory surface hopping simulations.

Figure 5a presents the CNNC dihedral angles during the dynamics process to illustrate how light–matter interactions affect the reaction ratio of the azomethane molecule, with the light–matter coupling strength *g*_c_ = 0.005 au. In this figure, we print out the event of the hopping from the |⟩ polariton state to the |⟩ polariton state. Here, we randomly selected 100 trajectories out of the 500 trajectories we generated in the simulation. As opposed to the case of outside the cavity (Figure 1d) where essentially no trajectory appears around the 0° CNNC dihedral angle, when coupling to the cavity, more trajectories are trapped around the *cis* isomer (Figure 5a) located at the 0° CNNC dihedral angle, and the CNNC dihedral angles of these trajectories oscillate centered at 0°. This indicates that fewer trajectories will go to the *trans* configuration, resulting in the suppression of the reaction ratio inside the cavity. Figure 5b presents the same results with a stronger light–matter coupling strength *g*_c_ = 0.05 au, where the fraction of trajectories trapped in the *cis* configuration region is further enhanced. The reason for the restricted motion of these trajectories is clear. After the trajectory leaves the Franck–Condon region, a large fraction of trajectories will evolve on the |⟩ state, as opposed to all moving to the electronic conical intersection between |*e*⟩ and |*g*⟩. Note that, although the NAC between the |⟩ state and |⟩ state is large (Figure 3d), not all trajectories will hop to the |⟩ state immediately when they approach the avoided crossing region between the  and |⟩ state. As shown in Figure 3a, there is a large energy barrier on the |⟩ polariton surface (violet curve), with the peak of the potential barrier located at the CNNC dihedral angle ∼90° (because it is largely the |*g*1⟩ state in that region). Hence, the rotation of these trajectories on the |⟩ surfaces from the *cis* to the *trans* configuration will be suppressed, and these trajectories are eventually forced back to the *cis* configuration region on the |⟩ surface. These trajectories will pass by the |⟩ and |⟩ avoided crossing region again and either hop to the |⟩ state or stay on the |⟩ surface. Eventually, they will decay to the |⟩ state around the *cis* isomer due to the cavity loss, giving rise to the hopping event indicated as the open circles in Figure 5. Note that there are many hops between the |⟩ and |⟩ surfaces as well. For clarity, we have not indicated these hopping events in the current plot.

**Figure 6:**
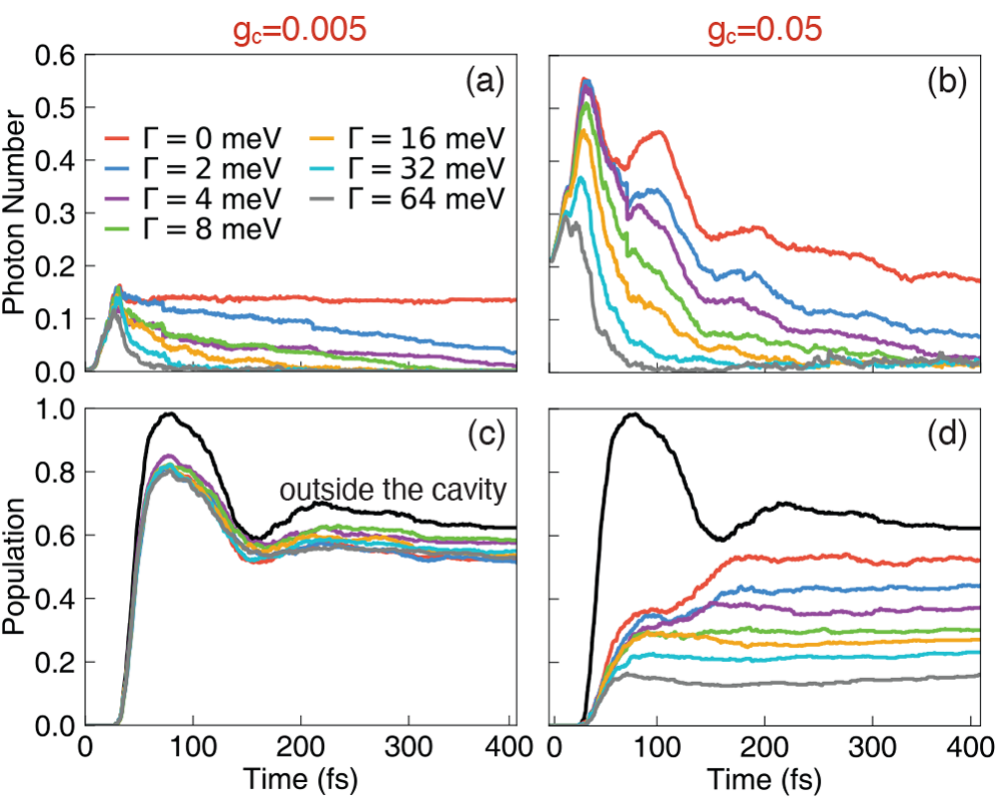
Polariton dynamics with changing cavity loss rate Γ. (a, b) The time-dependent photon number dynamics and (c, d) population of the *trans* isomer in the QED simulation with various cavity loss rates Γ. The light–matter coupling strengths are *g*_c_ = 0.005 au (in (a) and (c)) and *g*_c_ = 0.05 au (in (b) and (d)), respectively. The field is polarized along the *Y*-axis. The cavity frequency is set to ℏω_c_ = 2.72 eV. The black lines in panels (c) and (d) are the results outside the cavity, whereas the results inside the cavity with various Γ are color-coded using the legend inside panel (a).

Figure 6 presents the influence of the cavity loss rate Γ on average photon numbers (panels a and b) as well as the population of the *trans* isomer (panels c and d), with two different light–matter coupling strengths *g*_c_ = 0.005 au (left column) and *g*_c_ = 0.05 au (right column). For realistic molecule–cavity hybrid systems, due to the interaction between the cavity mode and the far-field photon modes outside the cavity, the lifetime of the cavity is always finite. In this work, we use the Lindblad dynamics to incorporate cavity loss in the TSH dynamics. To investigate how cavity loss can impact the chemical reaction, we perform the dynamics inside the cavity with different cavity loss rates Γ. For two coupling cases where *g*_c_ = 0.005 au and *g*_c_ = 0.05 au, the average photon number decreases when the cavity loss rate Γ increases, as clearly shown in Figure 6a and b.

Moreover, when the cavity loss rate increases, we have observed a trend of suppression of the reaction from the *cis* isomer to the *trans* isomer, resulting in a decreasing population of the *trans* isomer with increasing Γ. This is clearly shown in Figure 6d, when the light–matter coupling strength *g*_c_ = 0.05 au is large. Normally, one would expect that, when the loss rate is much larger than the Rabi splitting, the hybrid system will go back to the weak coupling regime and one will no longer observe the Rabi splitting from spectroscopy measurements. For the reactivities of the molecule, one can also see an enhanced suppression of the forward reaction from the *cis* to the *trans* isomer. This is because the |⟩ exciton state very quickly decays to the ground polariton states, thus preventing the isomerization from happening in the excited states.

These numerical results could provide theoretical insights into the original Ebbesen experiments on photoisomerization.^8^ This means that, even for a lossy cavity, one can protect the molecule from being isomerized by coupling it to a resonant cavity, resulting in suppressed reactivities. Of course, we must emphasize that the Ebbesen experiments on photoisomerization^8^ are conducted under the collective coupling regime where at least 10^6^ molecules are coupled to the Fabry–Pérot cavity. The current simulation, on the other hand, is only conducted for the case of a single molecule strongly coupled to the cavity and is, thus, not directly connected to the Ebbesen experiments.^8^ Nevertheless, the current theoretical development is promising to perform ab initio on-the-fly simulations of many molecules coupled to the cavity and investigate the polariton dynamics under the collective regime, and we aim to explore this in the near future.

On the other hand, several experimental works have demonstrated a single molecule (emitter) coupled to a plasmonic cavity.^77,78^ In particular, Baumberg and co-workers have achieved a single methylene blue molecule strongly coupled to the plasmonic cavity, with the Rabi splitting up to Ω_R_ = 305 meV, and the cavity quality factor *Q* = ω_c_/Γ = 16, where ω_c_ = 1.88 eV (which is in resonance with the methylene blue in that experiment^78^) and with a Γ = 117.5 meV. All of the parameters we used in our simulations are consistent with these state-of-the-art experiments^78^ and should be able to be directly tested with the single molecule in the plasmonic cavity experiment. We should also note that, for the case of a plasmonic cavity coupled to a single molecule,^78^ the single-mode PF Hamiltonian might not be an appropriate model.^79−81^ A more desired approach is to use ab initio theory^80,81^ to describe both molecules and the plasmonic nanoparticles, due to the strong coupling between them and also because the “cavity mirror” is getting close enough to the molecule and its ab initio description becomes necessary.^81^

## 5 Conclusion

In this work, we perform the on-the-fly non-adiabatic dynamics simulation of a realistic molecular system coupled to the cavity. We extend the TSH method to simulate the quantum dynamics of coupled electronic–photonic–nuclear DOFs and use the accurate nuclear gradient expressions developed in our previous work.^25^ The cavity loss is described with the Lindblad super operator, ensuring proper treatment of both population decay and decoherence among states.^28^ During the polariton dynamics simulations, the energies and gradients of the electronic states as well as the NAC between them are directly obtained from the *ab initio* on-the-fly electronic structure calculations at the level of CASSCF, while the molecular dipoles (including permanent and transition dipoles) and their derivatives are obtained from the machine learning model which is also trained with the data at the CASSCF level.

In the construction of the machine learning model of dipoles, we employ the KRR approach and the Coulomb matrix as the molecular descriptor to represent the molecular geometry. We define a relative Cartesian coordinate system (as opposed to the actual Cartesian coordinates of the molecule) in the machine learning process. Similar to the Coulomb matrix, dipoles can also be invariant to the translational and rotational motion of molecules based on the relative Cartesian coordinate system. The relative Cartesian coordinate system defined in this work provides the possibility to fit a molecular property that is dependent on the orientation of the molecular geometry. In the training process, we use the hierarchical agglomerative clustering algorithm, which largely reduces the training data set as well as the testing error.

We use azomethane as an example to investigate its photoisomerization reaction inside the cavity. We present the non-adiabatic dynamics simulations outside the cavity for comparison. The results show that the machine learning model works well in the prediction of molecular dipoles and derivatives. In addition, we observe that the reaction ratio of azomethane from the *cis* configuration to the *trans* configuration can be well controlled by tuning the light–matter coupling strengths, the polarized directions of the electric field (see additional results in the Supporting Information), and the cavity loss rates. We further provide mechanistic insight into how a cavity can modify photochemical reactivities by carefully analyzing the population dynamics, the cavity loss process, and the time evolution of the key nuclear degrees of freedom (the CNNC dihedral angle). We envision our theoretical results may provide new inspiration for the experimental investigations on polariton photochemistry in molecule–cavity hybrid systems.

The work demonstrates the powerful role of machine learning techniques in the on-the-fly *ab initio* polariton dynamics. By using the machine learning model in this work, if one electronic structure calculation method can provide the molecular dipoles, we can in principle employ it in the polariton dynamics simulations using any MQC methods. This opens the possibility of simulating the polariton dynamics with the excited-state electronic structure calculation methods that can treat more complex molecular systems, such as the algebraic diagrammatic construction method to the second order [ADC(2)]^82,83^ or time-dependent density functional theory (TD-DFT).^84,85^

Finally, although only a single molecule and a single cavity mode are considered in this work, we aim to extend the machine learning model to simulate collective molecules inside the cavity with multiple cavity modes if we can get all the molecular properties (energies, dipoles, NACs) with machine learning techniques. Simulating polariton dynamics under the collective coupling regime is one of the most ideal cases to take advantage of machine learning parametrization, because the massive training data will be worthwhile if one needs to carry out the dynamics of many chemically identical molecules collectively coupled to the cavity. This will be the future direction of the current work. We envision that the machine learning model for construction dipoles and their derivatives presented in this work will benefit the quantum dynamics community aiming at using *ab initio* simulations to investigate polariton dynamics in realistic molecule–cavity hybrid systems.

## Supporting Information Available

The details of the relative Cartesian coordinate system; the distribution of CNNC dihedral angles in the training data set; additional data for the test error in the KRR process; the derivation of the photon number operator in the dipole gauge; polariton potential energy surfaces with different Hamiltonian models; additional polariton dynamics results with the field polarized along the *Z*-axis; discussions on the energy conservation; Cartesian coordinates for the optimized *cis* isomer, *trans* isomer, and the conical intersection geometries. The source code for the polariton surface hopping dynamics and the source code for the machine learning, along with an example of training the dipole moment, are provided in a GitHub repository.^57^ (PDF)
