# Supplementary material for: Ab Initio Molecular Cavity Quantum Electrodynamics Simulations Using Machine Learning Models
Source: J Chem Theory Comput. 2023 Mar 31;19(8):2353–68. doi: 10.1021/acs.jctc.3c00137 (PMC10134431; doi:10.1021/acs.jctc.3c00137)
Supplement: Supplementary file 1 — ct3c00137_si_001.pdf [file ct3c00137_si_001.pdf]

# Supporting Information for
# Ab-initio Molecular Cavity Quantum Electrodynamics Simulations Using Machine Learning Models

Deping Hu*

*Department of Chemistry, University of Rochester,*

*120 Trustee Road, Rochester, NY 14627, U.S.A.*

Pengfei Huo†

*Department of Chemistry, University of Rochester,*

*120 Trustee Road, Rochester, NY 14627, U.S.A. and*

*The Institute of Optics, Hajim School of Engineering,*

*University of Rochester, Rochester, New York, 14627, U.S.A.*

## I. RELATIVE CARTESIAN COORDINATE SYSTEM

The definition of the relative Cartesian coordinate system is shown in Figure S1. We choose three different atoms (A, B and C) of the molecule as the reference atoms. For the azomethane molecule studied in this work, A and B are the two Nitrogen atoms, and C is one of the Carbon atoms. Then X-axis of the relative Cartesian coordinate system is defined along the $A \rightarrow B$ direction, the Y-axis is defined perpendicular to the plane formed by ABC, and the Z-axis is defined perpendicular to the X- and Y-axes simultaneously. We emphasize that we need to define individual relative coordinate systems for each of the conformers in the training dataset, since the positions of A, B and C atoms could vary for different conformers. Nevertheless, by using these three atoms, we uniquely define the 3 axes.

After we define the relative Cartesian coordinate system, we further transform the dipole based on the original Cartesian coordinate system in space (referred to as the global Cartesian coordinate system in this work) to the newly-defined relative Cartesian coordinate system. Below, we present details of the coordinate system transformation.

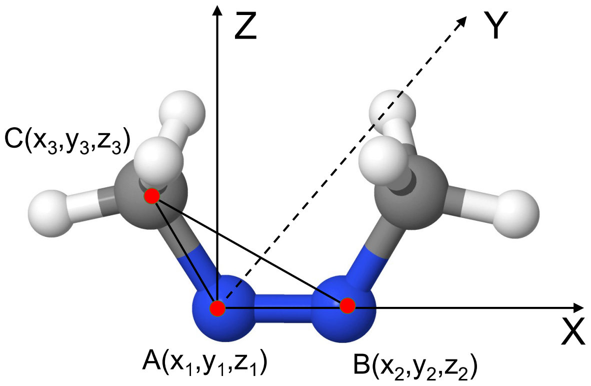


FIG. S1: Relative Cartesian coordinate system defined in this work. A, B and C are three reference atoms of the molecular system. For the azomethane molecule, A and B are the two Nitrogen atoms, and C is one of the Carbon atoms. For the relative Cartesian coordinate system, the X-axis is defined along the $A \rightarrow B$ direction, the Y-axis is defined perpendicular to the plane formed by ABC, and the Z-axis is defined perpendicular to the X- and Y-axes simultaneously.

The first step is to obtain the unit vectors along the X-, Y- and Z-axis directions ($\vec{x}$, $\vec{y}$

and $\vec{z}$), expressed as follows

$$\vec{x} = (a_1, b_1, c_1) \tag{S1a}$$

$$\vec{y} = (a_2, b_2, c_3) \tag{S1b}$$

$$\vec{z} = (a_3, b_3, c_3) \,. \tag{S1c}$$

Here, we already know the positions of A, B and C based on the global Cartesian coordinate system, which are $(x_1, y_1, z_1)$, $(x_2, y_2, z_2)$ and $(x_3, y_3, z_3)$, respectively. Since the X-axis is along the $A \rightarrow B$ direction, we can express $\vec{x}$ with its coordinate as

$$a_1 = \frac{(x_2 - x_1)}{\sqrt{\left((x_2 - x_1)^2 + (y_2 - y_1)^2 + (z_2 - z_1)^2\right)}} \tag{S2a}$$

$$b_1 = \frac{(y_2 - y_1)}{\sqrt{\left((x_2 - x_1)^2 + (y_2 - y_1)^2 + (z_2 - z_1)^2\right)}} \tag{S2b}$$

$$c_1 = \frac{(z_2 - z_1)}{\sqrt{\left((x_2 - x_1)^2 + (y_2 - y_1)^2 + (z_2 - z_1)^2\right)}}. \tag{S2c}$$

Next, considering the Y-axis is perpendicular to the plane formed by ABC, we can write the equation for the plane formed by ABC as $mx + ny + kz + l = 0$, where

$$\begin{aligned} m &= y_1 (z_2 - z_3) + y_2 (z_3 - z_1) + y_3 (z_1 - z_2) \\ n &= z_1 (x_2 - x_3) + z_2 (x_3 - x_1) + z_3 (x_1 - x_2) \\ k &= x_1 (y_2 - y_3) + x_2 (y_3 - y_1) + x_3 (y_1 - y_2) \\ l &= -x_1 (y_2 z_3 - y_3 z_2) - x_2 (y_3 z_1 - y_1 z_3) - x_3 (y_1 z_2 - y_2 z_1) \,. \end{aligned} \tag{S3a}$$

We can further express $\vec{y}$ using the coordinates as

$$a_2 = \frac{m}{\sqrt{m^2 + n^2 + k^2}} \tag{S4a}$$

$$b_2 = \frac{n}{\sqrt{m^2 + n^2 + k^2}} \tag{S4b}$$

$$c_2 = \frac{k}{\sqrt{m^2 + n^2 + k^2}}. \tag{S4c}$$

Finally, the Z-axis is perpendicular to the X- and Y-axes simultaneously, which means that $\vec{z} = \vec{x} \times \vec{y}$, resulting in

$$a_3 = b_1 c_2 - b_2 c_1 \tag{S5a}$$

$$b_3 = a_2 c_1 - a_1 c_2 \tag{S5b}$$

$$c_3 = a_1 b_2 - a_2 b_1. \tag{S5c}$$

Until now, we get the unit vectors of the axes of the relative Cartesian system. Next, we need to project the dipole to the new axes. Given the dipole based on the global Cartesian coordinate system as

$$\vec{\mu}^g = \left(\mu_x^g, \mu_y^g, \mu_z^g\right), \tag{S6}$$

where the notation $g$ denotes the dipole based on the global Cartesian coordinate system. Assuming the dipole based on the relative Cartesian coordinate system is

$$\vec{\mu}^r = \left(\mu_x^r, \mu_y^r, \mu_z^r\right), \tag{S7}$$

which can be obtained through the following projection equation

$$\mu_x^r = \vec{\mu}^g \cdot \vec{x} = \mu_x^g a_1 + \mu_y^g b_1 + \mu_z^g c_1 \tag{S8a}$$

$$\mu_y^r = \vec{\mu}^g \cdot \vec{y} = \mu_x^g a_2 + \mu_y^g b_2 + \mu_z^g c_2 \tag{S8b}$$

$$\mu_z^r = \vec{\mu}^g \cdot \vec{z} = \mu_x^g a_3 + \mu_y^g b_3 + \mu_z^g c_3. \tag{S8c}$$

We can further write Eq. S8 in a matrix representation as

$$\begin{pmatrix} \mu_x^r \\ \mu_y^r \\ \mu_z^r \end{pmatrix} = \begin{pmatrix} a_1 & b_1 & c_1 \\ a_2 & b_2 & c_2 \\ a_3 & b_3 & c_3 \end{pmatrix} \begin{pmatrix} \mu_x^g \\ \mu_y^g \\ \mu_z^g \end{pmatrix}. \tag{S9}$$

In this work, we use $\vec{\mu}^r$ instead of $\vec{\mu}^g$ in the training process. In the prediction process, since the predicated value is also $\vec{\mu}^r$, we need to transform it back to $\vec{\mu}^g$ using the following equation

$$\begin{pmatrix} \mu_x^g \\ \mu_y^g \\ \mu_z^g \end{pmatrix} = \begin{pmatrix} d_1 & e_1 & f_1 \\ d_2 & e_2 & f_2 \\ d_3 & e_3 & f_3 \end{pmatrix} \begin{pmatrix} \mu_x^r \\ \mu_y^r \\ \mu_z^r \end{pmatrix}, \tag{S10}$$

where

$$\begin{pmatrix} d_1 & e_1 & f_1 \\ d_2 & e_2 & f_2 \\ d_3 & e_3 & f_3 \end{pmatrix} = \begin{pmatrix} a_1 & b_1 & c_1 \\ a_2 & b_2 & c_2 \\ a_3 & b_3 & c_3 \end{pmatrix}^{-1} = \frac{1}{q} \begin{pmatrix} b_2c_3 - c_2b_3 & c_1b_3 - b_1c_3 & b_1c_2 - c_1b_2 \\ c_2a_3 - a_2c_3 & a_1c_3 - c_1a_3 & a_2c_1 - a_1c_2 \\ a_2b_3 - b_2a_3 & b_1a_3 - a_1b_3 & a_1b_2 - a_2b_1 \end{pmatrix}, \tag{S11}$$

and

$$q = a_1\left(b_2c_3 - c_2b_3\right) - a_2\left(b_1c_3 - c_1b_3\right) + a_3\left(b_1c_2 - c_1b_2\right). \tag{S12}$$

Using Eq. S10, we obtain

$$\mu_x^g = \mu_x^r d_1 + \mu_y^r e_1 + \mu_z^r f_1$$
$$\mu_y^g = \mu_x^r d_2 + \mu_y^r e_2 + \mu_z^r f_2$$
$$\mu_z^g = \mu_x^r d_3 + \mu_y^r e_3 + \mu_z^r f_3. \tag{S13a}$$

The derivative of the dipole on one of the nuclear DOF $R_i^g$ based on the global Cartesian coordinate is expressed as follows

$$\frac{\partial \mu_x^g}{\partial R_i^g} = \frac{\partial \mu_x^r}{\partial R_i^g} d_1 + \mu_x^r \frac{\partial d_1}{\partial R_i^g} + \frac{\partial \mu_y^r}{\partial R_i^g} e_1 + \mu_y^r \frac{\partial e_1}{\partial R_i^g} + \frac{\partial \mu_z^r}{\partial R_i^g} f_1 + \mu_z^r \frac{\partial f_1}{\partial R_i^g} \tag{S14a}$$
$$\frac{\partial \mu_y^g}{\partial R_i^g} = \frac{\partial \mu_x^r}{\partial R_i^g} d_2 + \mu_x^r \frac{\partial d_2}{\partial R_i^g} + \frac{\partial \mu_y^r}{\partial R_i^g} e_2 + \mu_y^r \frac{\partial e_2}{\partial R_i^g} + \frac{\partial \mu_z^r}{\partial R_i^g} f_2 + \mu_z^r \frac{\partial f_2}{\partial R_i^g} \tag{S14b}$$
$$\frac{\partial \mu_z^g}{\partial R_i^g} = \frac{\partial \mu_x^r}{\partial R_i^g} d_3 + \mu_x^r \frac{\partial d_3}{\partial R_i^g} + \frac{\partial \mu_y^r}{\partial R_i^g} e_3 + \mu_y^r \frac{\partial e_3}{\partial R_i^g} + \frac{\partial \mu_z^r}{\partial R_i^g} f_3 + \mu_z^r \frac{\partial f_3}{\partial R_i^g}. \tag{S14c}$$

We can also re-express the above expression (Eq. S14) in a matrix representation as

$$\begin{pmatrix} \frac{\partial \mu_x^g}{\partial R_i^g} \\ \frac{\partial \mu_y^g}{\partial R_i^g} \\ \frac{\partial \mu_z^g}{\partial R_i^g} \end{pmatrix} = \begin{pmatrix} d_1 & e_1 & f_1 \\ d_2 & e_2 & f_2 \\ d_3 & e_3 & f_3 \end{pmatrix} \begin{pmatrix} \frac{\partial \mu_x^r}{\partial R_i^g} \\ \frac{\partial \mu_y^r}{\partial R_i^g} \\ \frac{\partial \mu_z^r}{\partial R_i^g} \end{pmatrix} + \begin{pmatrix} \frac{\partial d_1}{\partial R_i^g} & \frac{\partial e_1}{\partial R_i^g} & \frac{\partial f_1}{\partial R_i^g} \\ \frac{\partial d_2}{\partial R_i^g} & \frac{\partial e_2}{\partial R_i^g} & \frac{\partial f_2}{\partial R_i^g} \\ \frac{\partial d_3}{\partial R_i^g} & \frac{\partial e_3}{\partial R_i^g} & \frac{\partial f_3}{\partial R_i^g} \end{pmatrix} \begin{pmatrix} \mu_x^r \\ \mu_y^r \\ \mu_z^r \end{pmatrix}. \tag{S15}$$

On the right-hand side of Eq. S15, the transformation matrix between the global Cartesian coordinate and the relative Cartesian coordinate can be obtained through Eq. S11. The derivative of the transformation matrix can be obtained by expanding Eq. S11 in terms of the global Cartesian coordinate. The dipoles can be obtained through the machine learning model. The analytical derivatives of the dipoles based on the "relative Cartesian coordinate", $\{\frac{\partial \mu_x^r}{\partial R_i^g}, \frac{\partial \mu_y^r}{\partial R_i^g}, \frac{\partial \mu_z^r}{\partial R_i^g}\}$ can be autograded from the Scikit-learn (a python toolbox). In principle, the analytical expression of the derivative of the transformation matrix in terms of the global Cartesian coordinate, such as $\frac{\partial d_1}{\partial R_i^g}$, can be derived with the SymPy[1] library of Python. However, the expression is extremely tedious and it is actually time-consuming to apply the analytical expression to get the derivative in the dynamics process.

A practical way is to get these derivatives numerically, with the following expression (take $\frac{\partial d_1}{\partial R_i^g}$ for example)

$$\frac{\partial d_1(R_i^g)}{\partial R_i^g} = \frac{d_1(R_i^g + \delta R_i^g) - d_1(R_i^g - \delta R_i^g)}{2\delta R_i^g}. \tag{S16}$$

In our model, $R_i^g$ is set to be 0.001 a.u. to get the converged results. Note that these numerical derivatives are only applied to evaluate the derivatives related to the coordinate

transformations, such as $\frac{\partial d_1(R_i^g)}{\partial R_i^g}$, and all derivatives related to the dipoles over the "relative Cartesian coordinate", $\{\frac{\partial \mu_x^r}{\partial R_i^g}, \frac{\partial \mu_y^r}{\partial R_i^g}, \frac{\partial \mu_z^r}{\partial R_i^g}\}$ are autograded from the Scikit-learn (a python toolbox). This is what we used in the actual numerical implementation.

## II. DISTRIBUTION OF THE CNNC DIHEDRAL ANGLES IN THE TRAINING DATASET

The distribution of the CNNC dihedral angles for azomethane in the final training dataset is shown in Fig. S2. We can see that the dihedral angles are well distributed (although not uniformly) from -180 to 180 degrees. Thus, the training dataset is properly sampled in this work.

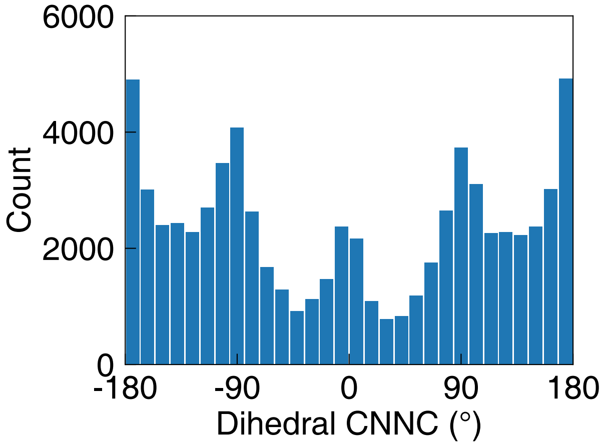


FIG. S2: Distributions of the CNNC dihedral angles in the training dataset.

## III. KERNEL RIDGE REGRESSION ERROR

To check the accuracy of the machine learning dipoles constructed in this work, we plot the distribution of the test errors in the learning procedure, as shown in Fig. S3. For the azomethane molecule studied in this work, there are two kinds of permanent dipoles (ground and excited states permanent dipoles) and one transition dipole (ground to excited state transition dipole), and each dipole has three components in different directions (X, Y, Z). Thus, we have nine different dipole components and we trained and tested them separately.

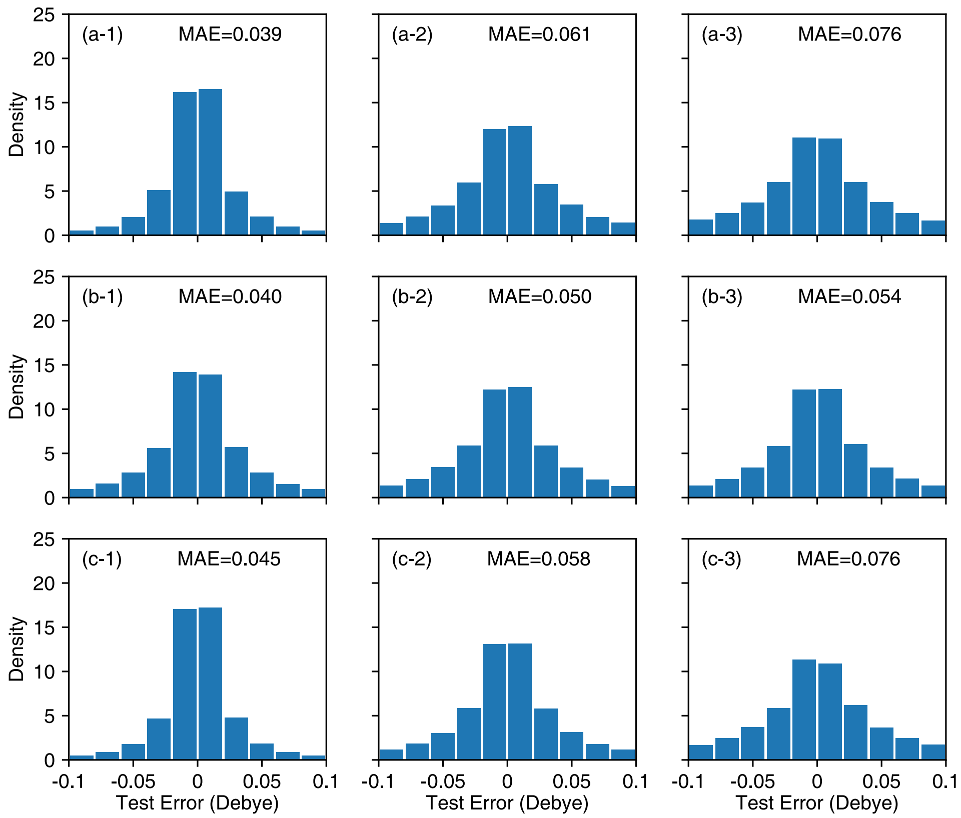


FIG. S3: Distributions of the test errors in the machine learning model for (a) permanent dipole of ground state, (b) transition dipole between ground state and excited state, and (c) permanent dipole of excited state, in different directions: (1) X-axis (left column), (2) Y-axis (middle column) and (3) Z-axis (right column).

Fig. S3 shows that the Kernel ridge regression method can well predict the permanent and transition dipoles for all components in different directions, with small mean average errors.

## IV. POLARITON POTENTIAL ENERGY SURFACE WITH DIFFERENT HAMILTONIANS

In the investigations of polariton photochemistry, besides the Pauli-Fierz (PF) Hamiltonian used in this work, several other Hamiltonian models have been used to describe the quantum light-matter interactions in previous studies, for example, the Jaynes-Cummings (JC) model[2,3] and Rabi model.[4] Next we will derive these models from the PF model Hamil-

tonian. We write the PF Hamiltonian below for convenience,

$$\begin{aligned}\hat{H}^{\mathrm{PF}} &= \hat{T}_{\mathrm{n}} + \hat{H}_{\mathrm{en}} + \hat{H}_{\mathrm{p}} + \hat{H}_{\mathrm{enp}} + \hat{H}_{\mathrm{d}} \\ &= \hat{T}_{\mathrm{n}} + \hat{H}_{\mathrm{en}} + \hat{H}_{\mathrm{p}} + g_{\mathrm{c}}\boldsymbol{\epsilon}\cdot\hat{\boldsymbol{\mu}}(\hat{a}^{\dagger}+\hat{a}) + \frac{g_{\mathrm{c}}^{2}}{\hbar\omega_{\mathrm{c}}}(\boldsymbol{\epsilon}\cdot\hat{\boldsymbol{\mu}})^{2}. \end{aligned} \tag{S17}$$

Take the azomethane molecule studied in this work as an example, only two electronic states $\{|g\rangle, |e\rangle\}$ are considered during the dynamics, the transition dipole between them is defined as $\boldsymbol{\mu}_{ge} = \langle g|\hat{\boldsymbol{\mu}}|e\rangle$. If we ignore the permanent dipole moments (PDMs) and further define the creation and annihilation operators for molecular excitation as $\hat{\sigma}^{\dagger} \equiv |e\rangle\langle g|$ and $\hat{\sigma} \equiv |g\rangle\langle e|$, the dipole operator can be written as $\hat{\boldsymbol{\mu}} = \boldsymbol{\mu}_{\mathrm{eg}}\cdot(\hat{\sigma}^{\dagger}+\hat{\sigma})$. The molecule-cavity interaction term can now be expressed as

$$\hat{H}_{\mathrm{enp}} = g_{\mathrm{c}}\boldsymbol{\epsilon}\cdot\boldsymbol{\mu}_{\mathrm{eg}}\cdot(\hat{a}^{\dagger}+\hat{a})(\hat{\sigma}^{\dagger}+\hat{\sigma}). \tag{S18}$$

If we further drop the dipole self-energy (DSE) term $\hat{H}_{\mathrm{d}}$, we can obtain the Rabi model as

$$\hat{H}^{\mathrm{Rabi}} = \hat{T}_{\mathrm{n}} + \hat{H}_{\mathrm{en}} + \hat{H}_{\mathrm{p}} + g_{\mathrm{c}}\boldsymbol{\epsilon}\cdot\boldsymbol{\mu}_{\mathrm{eg}}\cdot(\hat{a}^{\dagger}+\hat{a})(\hat{\sigma}^{\dagger}+\hat{\sigma}). \tag{S19}$$

Assuming the rotation wave approximation by ignoring the counter-rotating terms $\hat{a}^{\dagger}\hat{\sigma}^{\dagger}$ and $\hat{a}\hat{\sigma}$, we can arrive at the JC model,

$$\hat{H}^{\mathrm{JC}} = \hat{T}_{\mathrm{n}} + \hat{H}_{\mathrm{en}} + \hat{H}_{\mathrm{p}} + g_{\mathrm{c}}\boldsymbol{\epsilon}\cdot\boldsymbol{\mu}_{\mathrm{eg}}\cdot(\hat{a}^{\dagger}\hat{\sigma}+\hat{a}\hat{\sigma}^{\dagger}). \tag{S20}$$

Here we introduce two other models for comparison, namely the PF Hamiltonian without the DSE term,

$$\hat{H}^{\mathrm{NDSE}} = \hat{T}_{\mathrm{n}} + \hat{H}_{\mathrm{en}} + \hat{H}_{\mathrm{p}} + g_{\mathrm{c}}\boldsymbol{\epsilon}\cdot\hat{\boldsymbol{\mu}}(\hat{a}^{\dagger}+\hat{a}), \tag{S21}$$

and the PF Hamiltonian without the PDM,

$$\hat{H}^{\mathrm{NPDM}} = \hat{T}_{\mathrm{n}} + \hat{H}_{\mathrm{en}} + \hat{H}_{\mathrm{p}} + g_{\mathrm{c}}\boldsymbol{\epsilon}\cdot\boldsymbol{\mu}_{\mathrm{eg}}\cdot(\hat{a}^{\dagger}+\hat{a})(\hat{\sigma}^{\dagger}+\hat{\sigma}) + \frac{g_{\mathrm{c}}^{2}}{\hbar\omega_{\mathrm{c}}}(\boldsymbol{\epsilon}\cdot\boldsymbol{\mu}_{\mathrm{eg}})^{2}. \tag{S22}$$

Until now, we have four additional Hamiltonian models, which are the Rabi model, JC model, PF model without PDM, and PF model without DSE. To investigate the roles of the DSE term and PDM play in the formation of the polaritonic states, we plot the potential energy surfaces (PESs) of the polaritonic states for azomethane molecule based on these models and compare them with that based on the PF model. The results with the field polarized along the Y-axis and Z-axis are presented in Fig. S4 and Fig. S5, respectively.

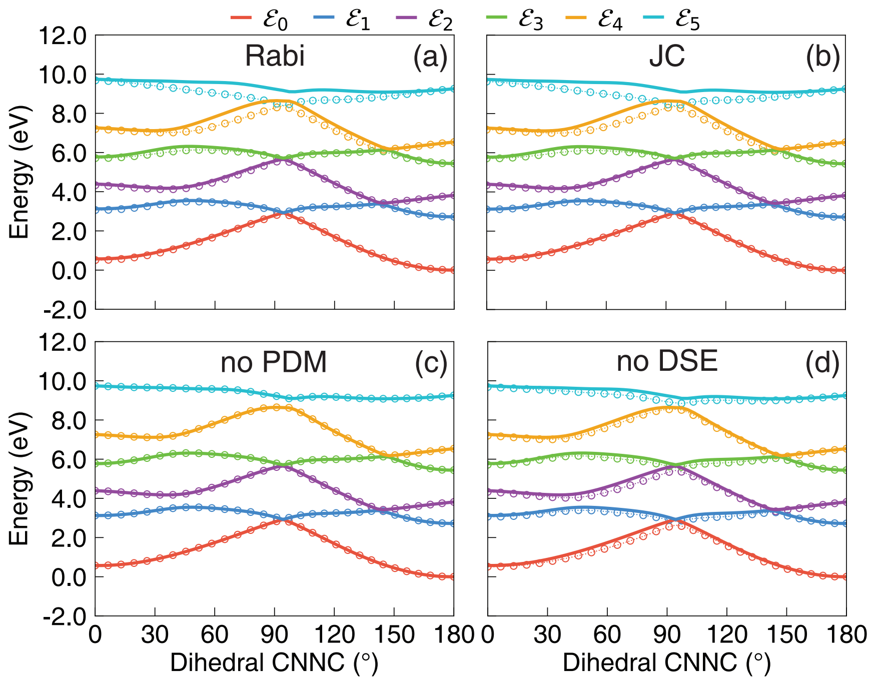


FIG. S4: Comparison of the PESs of the polaritonic states for azomethane molecule inside the cavity generated from the PF Hamiltonian (solid lines) with other four different Hamiltonian models (open circles): (a) Rabi model; (b) JC model; (c) PF model without the PDM; (d) PF model without the DSE. The light-matter coupling strength is $g_c = 0.05$ a.u. and the field is polarized along the Y-axis. The cavity frequency is set to $\hbar\omega_c = 2.72$ eV.

When the field is polarized along the Y-axis, the transition dipole is much larger than the permanent dipole, and the polariton PESs with different Hamiltonian models are shown in Fig. S4. For low-lying polaritonic states ($|\mathcal{E}_0\rangle$, $|\mathcal{E}_1\rangle$, $|\mathcal{E}_2\rangle$, $|\mathcal{E}_3\rangle$), the polariton PESs with all Hamiltonian models are in a good agreement. For high-lying polaritonic states ($|\mathcal{E}_4\rangle$, $|\mathcal{E}_5\rangle$), only the Rabi and JC models show obvious discrepancy compared to the PF Hamiltonian. Considering only some low-lying polaritonic states are populated during the dynamics for the molecular system studied in this work, we expect that polariton dynamics based on all the Hamiltonian models are similar in the case when the field is polarized along the Y-axis.

When the field is polarized along the Z-axis, the permanent dipole is much larger than the transition dipole, and the PESs with different Hamiltonian models are shown in Fig. S5. In this case, compared to the PESs obtained from the PF model, the PESs obtained from the other four Hamiltonian models (Rabi, JC, PF no PDM, PF no DSE) are much different. This large discrepancy is caused by large permanent dipoles for both $|g\rangle$ and $|e\rangle$ (see Fig. 2d

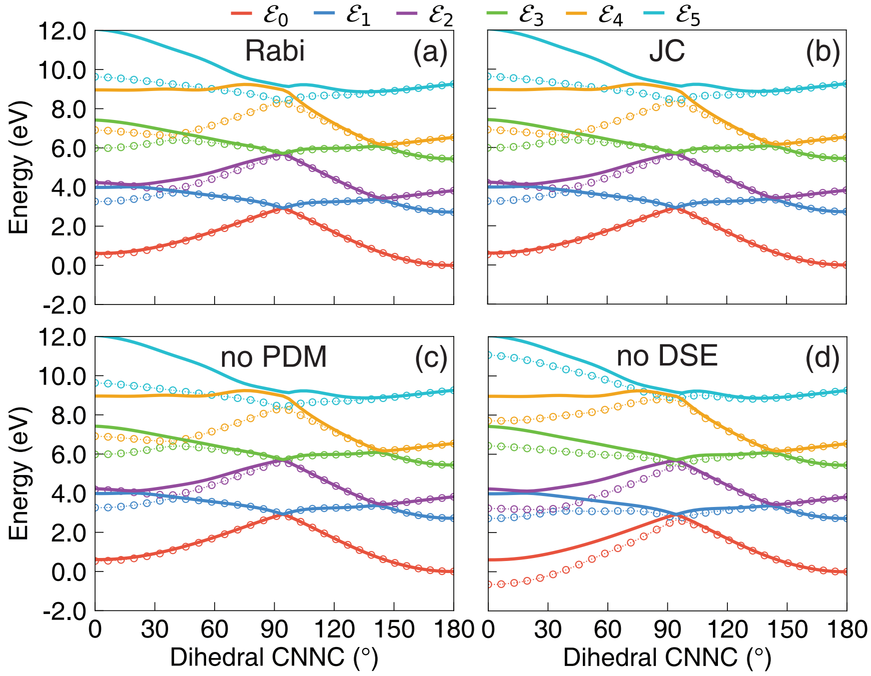


FIG. S5: Comparison of the polariton potential energy surface of the azomethane molecule coupled inside the cavity, obtained from the PF Hamiltonian (solid lines) with other four different Hamiltonian models (open circles), including (a) Rabi model; (b) JC model; (c) PF model without the PDM; (d) PF model without the DSE. The light-matter coupling strength is $g_c = 0.05$ a.u. and the field is polarized along the Z-axis. The cavity frequency is set to $\hbar\omega_c = 2.72$ eV.

of the main text). The light-matter interaction terms related to the permanent dipoles are completely ignored by the quantum optics model Hamiltonians, causing a large difference in polariton potentials compared to the full PH Hamiltonian treatment. As a result, the polariton dynamics should also be less accurate if we take these approximated Hamiltonian models instead of using the full PF model in the case when the field is polarized along the Z-axis.

## V. PHOTON NUMBER OPERATOR IN THE DIPOLE GAUGE

We provide a brief derivation of the "photon number" operator under the *dipole* gauge (the "d · E" form) used in the Pauli-Fierz (PF) Hamiltonian, where the details can be found in the related previous work.[5,6] Under the *Coulomb* gauge (the "p · A" form of light-matter

interactions), the photon number operator is expressed as

$$\hat{N}_{\text{p}\cdot\text{A}} = \hat{a}^\dagger\hat{a} = \frac{1}{2\hbar\omega_\text{c}}\hat{p}_\text{c}^2 + \frac{\omega_\text{c}}{2\hbar}\hat{q}_\text{c}^2 - \frac{1}{2}. \tag{S23}$$

Under the *dipole* gauge, the "photon number" operator should be

$$\begin{aligned}\hat{N}_{\text{d}\cdot\text{E}} &= \hat{U}\hat{a}^\dagger\hat{a}\hat{U}^\dagger = \hat{U}\hat{a}^\dagger\hat{U}^\dagger\hat{U}\hat{a}\hat{U}^\dagger \\ &= \frac{1}{2\hbar\omega_\text{c}}(\hat{p}_\text{c} + g_\text{c}\sqrt{\frac{2}{\hbar{\omega_\text{c}}^3}}\hat{\boldsymbol{\mu}}\cdot\boldsymbol{\epsilon})^2 + \frac{\omega_\text{c}}{2\hbar}\hat{q}_\text{c}^2 - \frac{1}{2},\end{aligned} \tag{S24}$$

where

$$\hat{U} = \exp\big[-\frac{i}{\hbar}\hat{\boldsymbol{\mu}}\cdot\boldsymbol{\epsilon}\frac{g_\text{c}}{{\omega_\text{c}}^2}\big(\hat{a} + \hat{a}^\dagger\big)\big], \tag{S25}$$

is the Power-Zienau-Woolley (PZW) Gauge transformation operator.[7,8] The "photon number" operator for the PF Hamiltonian can be obtained by using the following unitary transformation

$$\hat{N}_\text{PF} = \hat{U}_\phi\hat{N}_{\text{d}\cdot\text{E}}\hat{U}_\phi^\dagger = \frac{1}{2\hbar\omega_\text{c}}\hat{p}_\text{c}^2 + \frac{\omega_\text{c}}{2\hbar}(\hat{q}_\text{c} + g_\text{c}\sqrt{\frac{2}{\hbar{\omega_\text{c}}^3}}\hat{\boldsymbol{\mu}}\cdot\boldsymbol{\epsilon})^2 - \frac{1}{2}, \tag{S26}$$

where

$$\hat{U}_\phi = \exp[-i\frac{\pi}{2}\hat{a}^\dagger\hat{a}]. \tag{S27}$$

One can further express Eq. S26 as

$$\begin{aligned}\hat{N}_\text{PF} &= \frac{1}{2\hbar\omega_\text{c}}\hat{p}_\text{c}^2 + \frac{\omega_\text{c}}{2\hbar}(\hat{q}_\text{c} + g_\text{c}\sqrt{\frac{2}{\hbar{\omega_\text{c}}^3}}\hat{\boldsymbol{\mu}}\cdot\boldsymbol{\epsilon})^2 - \frac{1}{2} \\ &= \frac{1}{2\hbar\omega_\text{c}}\hat{p}_\text{c}^2 + \frac{\omega_\text{c}}{2\hbar}\hat{q}_\text{c}^2 + \frac{\omega_\text{c}}{2\hbar}\hat{q}_\text{c}g_\text{c}\sqrt{\frac{2}{\hbar\omega_\text{c}^3}}\hat{\boldsymbol{\mu}}\cdot\boldsymbol{\epsilon} + \frac{\omega_\text{c}}{2\hbar}g_\text{c}\sqrt{\frac{2}{\hbar\omega_\text{c}^3}}\hat{\boldsymbol{\mu}}\cdot\boldsymbol{\epsilon}\hat{q}_\text{c} + \frac{\omega_\text{c}}{2\hbar}(g_\text{c}\sqrt{\frac{2}{\hbar\omega_\text{c}^3}}\hat{\boldsymbol{\mu}}\cdot\boldsymbol{\epsilon})^2 - \frac{1}{2} \\ &= \hat{a}^\dagger\hat{a} + \frac{\omega_\text{c}}{2\hbar}\hat{q}_\text{c}g_\text{c}\sqrt{\frac{2}{\hbar\omega_\text{c}^3}}\hat{\boldsymbol{\mu}}\cdot\boldsymbol{\epsilon} + \frac{\omega_\text{c}}{2\hbar}g_\text{c}\sqrt{\frac{2}{\hbar\omega_\text{c}^3}}\hat{\boldsymbol{\mu}}\cdot\boldsymbol{\epsilon}\hat{q}_\text{c} + \frac{\omega_\text{c}}{2\hbar}(g_\text{c}\sqrt{\frac{2}{\hbar\omega_\text{c}^3}}\hat{\boldsymbol{\mu}}\cdot\boldsymbol{\epsilon})^2 \\ &= \hat{a}^\dagger\hat{a} + \frac{\omega_\text{c}}{2\hbar}\sqrt{\frac{\hbar}{2\omega_\text{c}}}(\hat{a}^\dagger + \hat{a})g_\text{c}\sqrt{\frac{2}{\hbar\omega_\text{c}^3}}\hat{\boldsymbol{\mu}}\cdot\boldsymbol{\epsilon} + \frac{\omega_\text{c}}{2\hbar}g_\text{c}\sqrt{\frac{2}{\hbar\omega_\text{c}^3}}\hat{\boldsymbol{\mu}}\cdot\boldsymbol{\epsilon}\sqrt{\frac{\hbar}{2\omega_\text{c}}}(\hat{a}^\dagger + \hat{a}) + \frac{\omega_\text{c}}{2\hbar}(g_\text{c}\sqrt{\frac{2}{\hbar\omega_\text{c}^3}}\hat{\boldsymbol{\mu}}\cdot\boldsymbol{\epsilon})^2 \\ &= \hat{a}^\dagger\hat{a} + \frac{g_\text{c}}{2\omega_\text{c}\hbar}(\hat{a}^\dagger + \hat{a})\hat{\boldsymbol{\mu}}\cdot\boldsymbol{\epsilon} + \frac{g_\text{c}}{2\omega_\text{c}\hbar}\hat{\boldsymbol{\mu}}\cdot\boldsymbol{\epsilon}(\hat{a}^\dagger + \hat{a}) + \frac{\omega_\text{c}}{2\hbar}(g_\text{c}\sqrt{\frac{2}{\hbar\omega_\text{c}^3}}\hat{\boldsymbol{\mu}}\cdot\boldsymbol{\epsilon})^2 \\ &= \hat{a}^\dagger\hat{a} + \frac{g_\text{c}}{\omega_\text{c}\hbar}\hat{\boldsymbol{\mu}}\cdot\boldsymbol{\epsilon}(\hat{a}^\dagger + \hat{a}) + \frac{g_\text{c}^2}{\omega_\text{c}^2\hbar^2}(\hat{\boldsymbol{\mu}}\cdot\boldsymbol{\epsilon})^2 \\ &= \hat{a}^\dagger\hat{a} + \frac{1}{\hbar\omega_\text{c}}(\hat{H}_\text{enp} + \hat{H}_\text{d}) \\ &= \frac{1}{\hbar\omega_\text{c}}(\hat{H}_\text{p} + \hat{H}_\text{enp} + \hat{H}_\text{d}) - \frac{1}{2}.\end{aligned}$$

## VI. QED SIMULATIONS WITH FIELD POLARIZED ALONG THE Z-AXIS

As pointed out in Sec. 4.2 in the main text, the magnitudes of the dipoles (permanent and transition dipoles) for the azomethane molecule in different directions vary a lot. To explore how the spatial orientation of the molecule affects the polariton dynamics, we also perform the QED simulation with field polarized along the Z-axis, which means $(\epsilon_x, \epsilon_y, \epsilon_z)$ is set to $(0, 0, 1)$ in Eq. 16 of the main text. Here, only the components of dipoles along the Z-axis contribute to the light-matter coupling. Same as the Y-polarized case, we consider two different light-matter coupling strengths: $g_\mathrm{c} = 0.005$ a.u. and $g_\mathrm{c} = 0.05$ a.u.

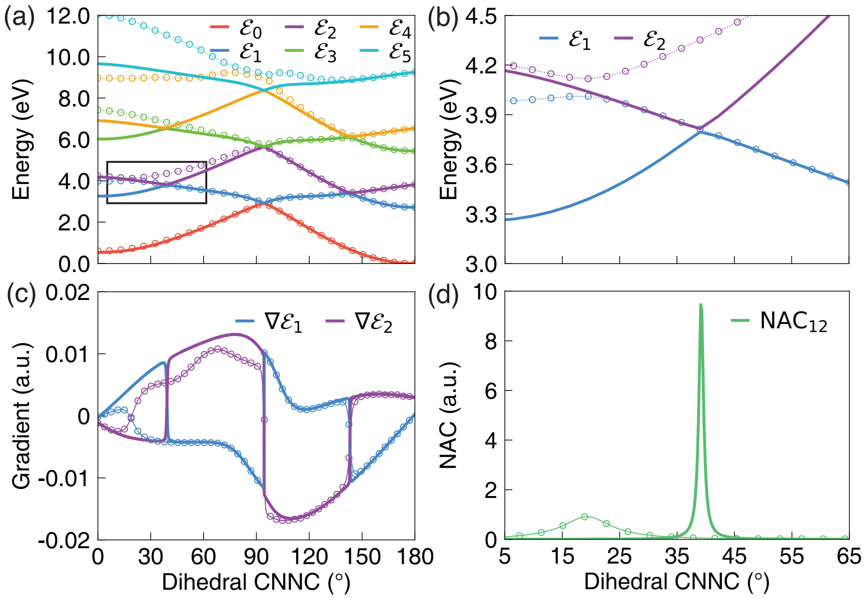


FIG. S6: The (a-b) PESs, (c) gradients and (d) NACs of the polaritonic states for azomethane molecule inside the cavity. The field is polarized along the Z-axis. The results obtained with the light-matter coupling strength $g_\mathrm{c} = 0.005$ a.u. and $g_\mathrm{c} = 0.05$ a.u. are plotted with solid lines and open circles, respectively. The cavity frequency is set to $\hbar\omega_\mathrm{c} = 2.72$ eV.

When using $g_\mathrm{c} = 0.005$ a.u., because the transition dipole along the Z-axis is small (Fig. 2d in the main text), the avoided crossing region between the $|\mathcal{E}_2\rangle$ and $|\mathcal{E}_1\rangle$ states becomes narrow with a very small Rabi splitting, as shown in Fig. S6a and Fig. S6b. Thus, the NAC between the $|\mathcal{E}_2\rangle$ and $|\mathcal{E}_1\rangle$ states (Fig. S6d) is much larger than that in the Y-polarized case (Fig. 3d in the main text). As a result, the polariton population transfers very fast from the $|\mathcal{E}_2\rangle$ state to the $|\mathcal{E}_1\rangle$ state at the beginning of the dynamics, see Fig. S7a. In addition, due to the small transition dipole, there is only a small amount of population transferred to the

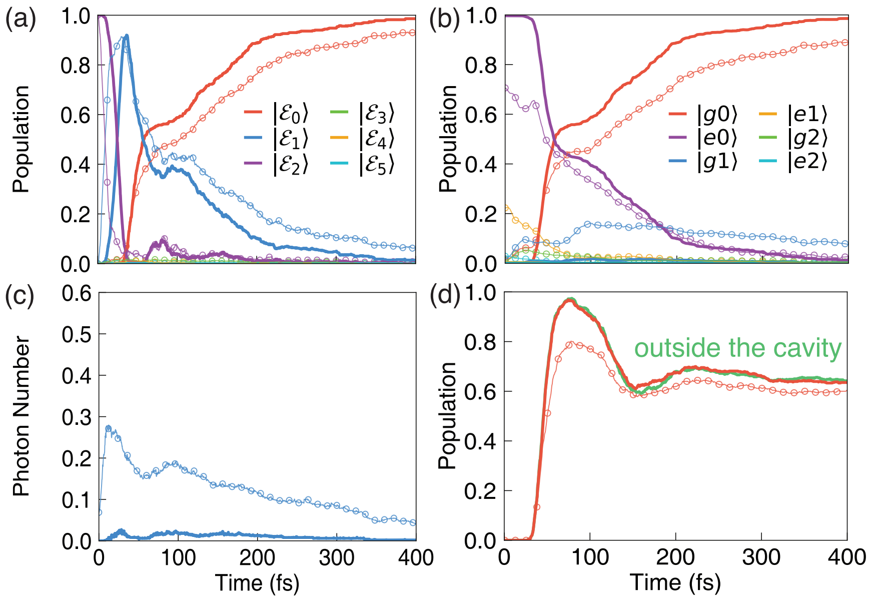


FIG. S7: The time-dependent properties in the QED simulation: (a) population of the polaritonic states; (b) population of the adiabatic-Fock states; (c) the average photon number population; (d) population of the *trans* isomer. The results obtained with the light-matter coupling strength $g_c = 0.005$ a.u. and $g_c = 0.05$ a.u. are plotted with solid lines and open circles, respectively. The cavity loss rate is set to $\Gamma = 4$ meV. The field is polarized along the Z-axis. The cavity frequency is set to $\hbar\omega_c = 2.72$ eV.

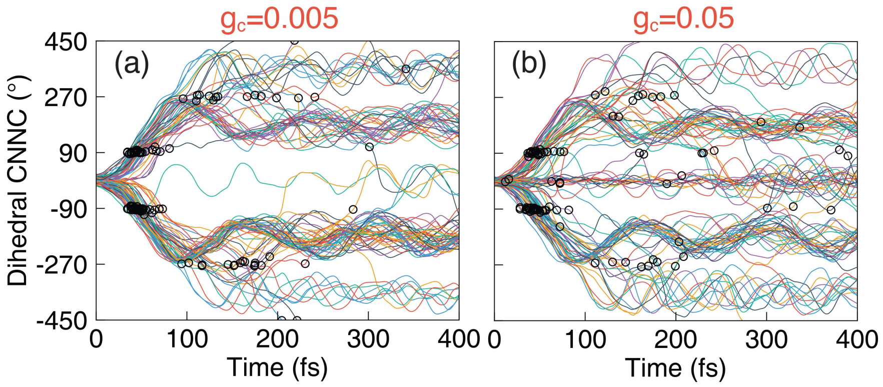


FIG. S8: The time-dependent values of the dihedral CNNC angles (of 100 trajectories) in the QED simulation for the azomethane molecule with light-matter coupling strength (a) $g_c = 0.005$ a.u. and (b) $g_c = 0.05$ a.u. The cavity loss rate is set to $\Gamma = 4$ meV. The field is polarized along the Z-axis. The cavity frequency is set to $\hbar\omega_c = 2.72$ eV. The black circles indicate where the hop events happen from the $|\mathcal{E}_1\rangle$ state to the $|\mathcal{E}_0\rangle$ state in the TSH dynamics.

$|g1\rangle$ state from $|e0\rangle$ state (Fig. S7b). The features of the polariton dynamics, such as the

population of the *trans* isomer (Fig. S7d) and the evolution of the dihedral CNNC angles (Fig. S8a) are also similar to those outside the cavity (Fig. 1c and 1d in the main text), due to the small dipole and light-matter coupling strength.

When increasing the coupling strength to $g_c = 0.05$ a.u., comparing to the Y-polarized case with the same light-matter coupling strength, the most obvious difference is that the avoided crossing between the $|\mathcal{E}_2\rangle$ and $|\mathcal{E}_1\rangle$ states shifts to the *cis* isomer site with a smaller dihedral CNNC angle, see Fig. S6a and b. This is because the permanent dipoles dominate the light-matter coupling in this case, and the permanent dipole of the $|g\rangle$ state is larger than that of the $|e\rangle$ state, as shown in Fig. 2d in the main text. The crossing between the gradients of the $|\mathcal{E}_2\rangle$ and $|\mathcal{E}_1\rangle$ states and the peak of the NAC between them also shift, see Fig. S6c and Fig. S6d. Since the energy gap between the $|\mathcal{E}_2\rangle$ and $|\mathcal{E}_1\rangle$ states is close to each other near the Franck-Condon region, the system transforms very fast (within 30 fs) from the $|\mathcal{E}_2\rangle$ state to the $|\mathcal{E}_1\rangle$ state, see the open circles in Fig. S7a. After that, the system stays at the $|\mathcal{E}_1\rangle$ state for a longer time than the $g_c = 0.005$ a.u. case, due to the energy barrier on the PES of the $|\mathcal{E}_1\rangle$ state (Fig. S6b).

The adiabatic-Fock state population (open circles in Fig. S7b) for $g_c = 0.05$ a.u. becomes complicated. First, near the Franck-Condon region, besides the $|e0\rangle$ state, the $|e1\rangle$ state also contributes significantly to the $|\mathcal{E}_2\rangle$ state (Fig. S7b) due to the large permanent dipole of the $|e\rangle$ state. Second, during the dynamics, the $|e1\rangle$ state can either transform to the $|g1\rangle$ state through the CI between the $|e\rangle$ and $|g\rangle$ states or transform to the $|e2\rangle$ state through the light-matter coupling caused by the permanent dipole of the $|e\rangle$ state. Third, the $|g1\rangle$ state can further transform to the $|g2\rangle$ state through the light-matter coupling caused by the permanent dipole of the $|g\rangle$ state. As a result, we can observe that the $|e2\rangle$, $|g2\rangle$, and $|g1\rangle$ states are populated during the dynamics, especially at the early time. All of the above states as well as the $|e1\rangle$ state contribute to the photon number evolution, as shown in Fig. S7c. The population of the *trans* isomer shows a slight decrease (Fig. S7d) when compared to the dynamics outside the cavity since more trajectories oscillate around the *cis* configuration (Fig. S8b) as the Y-polarized case.

## VII. DISCUSSIONS ON THE ENERGY CONSERVATION FOR POLARITON DYNAMICS SIMULATIONS

We want to discuss the energy conservation of our surface hopping simulation for the hybrid polariton systems. Below, we discuss three factors that could influence energy conservation during our simulation.

First, the gradient expressions (Eq. 33 and Eq. 34 of the main texts) are *exact* for the mixed quantum-classical system and will ensure energy conservation, if there is no cavity loss. This is because these nuclear gradient expressions for the polariton systems were derived by assuming the energy conservation of the mixed quantum-classical system (electron-photon as a quantum subsystem and nuclei as a classical subsystem). The derivation was detailed in Eq. 21-22 of Ref. 9. We have also carefully checked the energy conservation in model system calculations in Ref. 9

Second, in our KRR ML model, instead of training the derivative of the dipole moment directly, we train the dipole moment and then use the analytical expression to get the derivative of the dipole moment, which means the dipole moments and their derivatives are consistent with each other, ensuring the energy conservation when the exact nuclear gradient is used for the polariton hybrid system.

Third, for the CASSCF on-the-fly simulations, energy conservation highly depends on the molecular system. If for all nuclear configurations, the active space is sufficient to describe its excited states, then energy is well-conserved. For the system we investigated here, during the rotation of the molecule, the active space (six electrons in four orbitals) is large enough to describe the first two electronic states.

Fig. S9 presents the total energy (blue ) along with the polaritonic potential energy (red) and nuclear kinetic energy (green) during the polariton dynamics for a typical trajectory, with cavity loss as $\Gamma = 0$ meV (left) and $\Gamma = 4$ meV (right). Further, the purple lines indicate the current active state during the surface hopping simulation, with the active polariton state labeled on the right-hand side of each panel. For panel (a), it shows that the total energy is well conserved if the cavity loss rate is zero. Although the potential energy of the current active polaritonic state will suddenly jump during some regions due to the "hopping" of the system, we use the velocity scaling algorithm to modify the kinetic energy and make the total energy conserved before and after hopping.

For the finite cavity loss rate case in Fig. S9b, since we do not perform the velocity re-scaling if the hopping of the system is caused by the Lindblad dynamics (dressed by the photon decay process in Eq. 27b of the main text), the total energy is not conserved. In Fig. S9b, the total energy (blue line) decrease suddenly at $t \approx 200$ fs, when the hoping event happens between $|\mathcal{E}_1\rangle$ to $|\mathcal{E}_0\rangle$, where the energy is assumed to dissipate into the photonic environment due to the cavity loss.

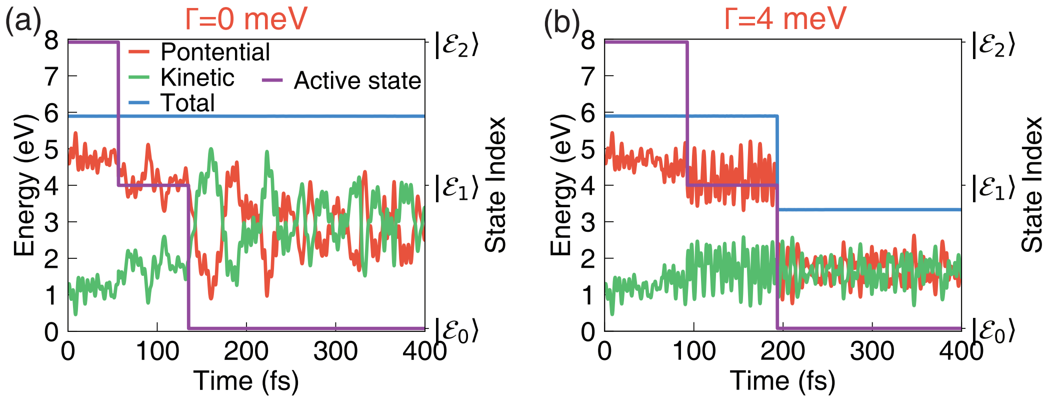


FIG. S9: The time-dependent potential energy (red line) associated with the current active polariton state, kinetic energy (green line) of the nuclei and the total energy (blue) of the system for typical trajectories during the TSH polariton dynamics. The purple line indicates the index of the current active state, which is labeled on the right-hand side of the Y-axis for each panel. The dynamics with the cavity loss rate Γ=0 meV (no cavity loss) and Γ=4 meV are presented in panel (a) and panel (b), respectively. The light-matter coupling strength is $g_c = 0.05$ a.u. The field is polarized along the Y-axis. The cavity frequency is set to $\hbar\omega_c = 2.72$ eV.

## VIII. CARTESIAN COORDINATES FOR SOME KEY CONFIGURATIONS

Ground-state minimum of the *Cis* isomer (optimized at the level of B3LYP/6-31G*)

| | | | |
|---|---|---|---|
| $N$ | 0.00000 | 0.00000 | 0.00000 |
| $N$ | 1.24304 | 0.00000 | 0.00000 |
| $C$ | −0.74229 | 0.00000 | −1.28432 |
| $C$ | 1.98532 | −0.00016 | −1.28433 |
| $H$ | −1.80740 | −0.00305 | −1.04833 |
| $H$ | −0.50818 | 0.89064 | −1.88202 |
| $H$ | −0.50359 | −0.88700 | −1.88558 |
| $H$ | 1.75109 | −0.89080 | −1.88199 |
| $H$ | 1.74669 | 0.88685 | −1.88561 |
| $H$ | 3.05043 | 0.00280 | −1.04835 |

Ground-state minimum of the *Trans* isomer (optimized at the level of B3LYP/6-31G*)

| | | | |
|---|---|---|---|
| $N$ | 0.00000 | 0.00000 | 0.00000 |
| $N$ | 1.24415 | 0.00000 | 0.00000 |
| $C$ | −0.56172 | 0.00000 | −1.35571 |
| $C$ | 1.80587 | 0.00005 | 1.35570 |
| $H$ | −1.20268 | 0.88407 | −1.45418 |
| $H$ | 0.21260 | −0.00114 | −2.13150 |
| $H$ | −1.20581 | −0.88148 | −1.45326 |
| $H$ | 1.03155 | 0.00122 | 2.13150 |
| $H$ | 2.44682 | −0.88402 | 1.45421 |
| $H$ | 2.44997 | 0.88153 | 1.45322 |

Conical intersection (optimized at the level of SA-2-CAS(6,4)/6-31G*)

| | | | |
|---|---|---|---|
| $N$ | 0.00000 | 0.00000 | 0.00000 |
| $N$ | 1.27667 | 0.00000 | 0.00000 |
| $C$ | −0.60803 | 0.00000 | −1.32884 |
| $C$ | 2.21900 | −1.07580 | 0.08071 |
| $H$ | −1.62890 | −0.34552 | −1.23458 |
| $H$ | −0.61980 | 1.01460 | −1.71124 |
| $H$ | −0.06100 | −0.62909 | −2.02451 |
| $H$ | 2.09467 | −1.62805 | 1.00824 |
| $H$ | 2.11270 | −1.76687 | −0.75369 |
| $H$ | 3.21377 | −0.65580 | 0.05607 |

* Electronic address: deping.hu@rochester.edu

† Electronic address: pengfei.huo@rochester.edu

[1] A. Meurer, C. P. Smith, M. Paprocki, O. Čertík, S. B. Kirpichev, M. Rocklin, A. Kumar, S. Ivanov, J. K. Moore, S. Singh, et al., PeerJ Computer Science **3**, e103 (2017), ISSN 2376-5992, URL https://doi.org/10.7717/peerj-cs.103.

[2] Y. Zhang, T. Nelson, and S. Tretiak, J. Chem. Phys. **151**, 154109 (2019).

[3] K. Bennett, M. Kowalewski, and S. Mukamel, Faraday Discuss. **194**, 259 (2016).

[4] A. F. Kockum, A. Miranowicz, S. D. Liberato, S. Savasta, and F. Nori, Nature Rev. Phys. **1**, 19 (2019).

[5] A. Mandal, S. M. Vega, and P. Huo, J. Phys. Chem. Lett. **11**, 9215 (2020).

[6] C. Schäfer, M. Ruggenthaler, V. Rokaj, and A. Rubio, ACS Photonics **7**, 975 (2020).

[7] E. A. Power and S. Zienau, Philos. Trans. Royal Soc. A **251**, 427 (1959).

[8] C. Cohen-Tannoudji, J. Dupont-Roc, and G. Grynberg, *Photons and Atoms: Introduction to Quantum Electrodynamics* (Wiley, 1997), ISBN 978-0-471-18433-1, URL https://www.ebook.de/de/product/3737960/claude_cohen_tannoudji_jacques_dupont_roc_gilbert_grynberg_photons_and_atoms_introduction_to_quantum_electrodynamics.html.

[9] W. Zhou, D. Hu, A. Mandal, and P. Huo, J. Chem. Phys. **157**, 104118 (2022).
